# Thrilling News Revisited: The Role of Suspense for the Enjoyment of News Stories

**DOI:** 10.3389/fpsyg.2016.01913

**Published:** 2016-12-15

**Authors:** Kai Kaspar, Daniel Zimmermann, Anne-Kathrin Wilbers

**Affiliations:** Department of Psychology, University of CologneCologne, Germany

**Keywords:** news perception, affective-disposition theory, suspense, enjoyment of narratives, personal relevance

## Abstract

Previous research on news perception has been dominated by a cognitively oriented perspective on reception processes, whereas emotions have been widely neglected. Consequently, it has remained open which features of a news story might elicit affective responses and hence modulate news perception, shifting the focus to the emotional potential of the narrative. According to the affective-disposition theory, the experience of suspense is the striving force of immersion in fictional dramas. Thereby, a positive affective disposition toward the protagonist of a story and a high likelihood of a bad ending should increase suspense that, in turn, should positively influence reading appreciation and lingering interest in the story. We investigated whether suspense and its determinants also play such a key role in the context of news stories. Study 1 (*n* = 263) successfully replicated results of an earlier study, whereas Studies 2 (*n* = 255) and 3 (*n* = 599) challenged the generalizability of some effects related to manipulated characteristics of a news story. In contrast, correlational relationships between perceived news characteristics and news evaluation were relatively stable. In particular, participants' liking of the protagonist and the perceived likelihood of a good ending were positively associated with suspense, reading appreciation, and lingering interest. This result indicates a preference for happy endings and contradicts the notion that likely negative outcomes are beneficial for suspense and the enjoyment of news stories, as postulated by the affective-disposition theory in the context of fictional dramas. Moreover, experienced suspense reliably mediated the correlations between, on the one hand, participants' liking of the protagonist and the perceived likelihood of a good ending and, on the other hand, reading appreciation and lingering interest. The news story's personal relevance was less influential than expected. Further, we observed a large absence of interaction effects, indicating that central characteristics of a news story can be independently varied to a large degree. In a nutshell, we may conclude that suspense significantly mediates the correlation between perceived news characteristics and the enjoyment of news stories, whereas manipulations of news characteristics do not necessarily influence the enjoyment of narratives as desired.

## Introduction

The distribution of information by means of news is a central process of human communication and society. Not surprisingly, the perception of news is one main topic of media research. Besides effects of peripheral cues (e.g., Gerend and Sias, 2009; Kaspar et al., 2015a) and the visualization of news (e.g., Kaspar et al., 2015c; Lee and Kim, 2015) on reception and evaluation processes, researchers intensively investigated how news influences perceived issue salience as well as attitudes and opinions (cf. McCombs and Reynolds, 2002; Reese, 2007). However, previous news research has established a cognitively oriented perspective on reception processes, whereas emotions—including discrete emotions, affect, and mood states—have been neglected in the context of news (for a detailed discussion about conceptual differences and similarities between emotions, affect, and mood, see Gray and Watson, 2001; Kaspar and König, 2012). In contrast, emotional processes have become a prevalent aspect of entertainment research (e.g., Vorderer et al., 2004; Jansz, 2005; Green and Clark, 2013). Accordingly, Knobloch-Westerwick and Keplinger (2007) resumed that “in general, news research implicitly considers media news and information to be a distinct form of communication, very much apart from other formats such as advertisements and entertainment” (p. 194). For example, the enjoyment of a narrative and its immersive potential are important for entertainment, but we may question whether one can similarly enjoy a news story about negative events (e.g., a tragic accident or kidnapping). Thus, this classic and seemingly plausible separation of information and entertainment has presumably distracted researchers' attention from emotional processes in news research for a long time. The present studies aim to bridge this conceptual and empirical gap by focusing on the critical role of suspense, defined as “a noxious affective reaction that characteristically derives from the respondents' acute, fearful apprehension about deplorable events that threaten liked protagonists, this apprehension being mediated by high but not complete subjective certainty about the occurrence of the anticipated deplorable events” (Zillmann, 1996, p. 208). In the following paragraphs, we will outline how emotional processes have been addressed by news research so far and why we should have a closer look to the emotional responses elicited by news stories, in particular suspense experiences.

Although a cognitively oriented perspective has been dominating news research on a large scale, some studies have already pursued an emotion-oriented perspective. Some of them examined how emotional states, induced prior news reception, alter the selection and memory for emotional news (e.g., Biswas et al., 1994; Kaspar et al., 2015b) as well as news evaluation (e.g., Wirth et al., 2010). Some other studies focused on the reception phase by investigating, inter alia, the impact of emotion-laden news images on the selection of news content from an internet news magazine when being in a browsing mode (e.g., Knobloch et al., 2003). Finally, some studies investigated the effects of emotional news content in the post-receptive phase (e.g., Brosius, 1993; Newhagen, 1998; Unz et al., 2008). These studies have in common that they focus on effects of emotional images or videos embedded in the news coverage. Similarly, some studies examined how the sequence of different emotion-laden television news affect recipients' evaluation of news content (Zillmann et al., 1994) or their affective reactions (Mundorf and Zillmann, 1991). However, in all studies of this line of research it still remains open which specific features of a news story may elicit specific emotional responses and hence modulate news perception, shifting the focus to the emotional potential of the narrative.

A complementary line of research examined how features of news stories are able to modulate recipients' emotional responses and news perception. These studies on framing effects examine how recipients' cognitive and emotional responses are affected by opposing ways of presenting, or framing, an event or issue (Gross and D'Ambrosio, 2004), while they typically refer to appraisal theories positing that emotional responses are a product of people's cognitive evaluation of event characteristics and stimuli (e.g., Ortony et al., 1988; Roseman, 1991). Indeed, several studies found that recipients' emotional responses were affected by the framing of news stories about victimization (Aust and Zillmann, 1996), social riots (Gross and D'Ambrosio, 2004), consequences of immigration (Igartua et al., 2011), policy support (Goodall et al., 2013), and traumatic events (Balzarotti and Ciceri, 2014). Of course, the relationship between cognitive appraisal and emotional responses is not a one-way street as emotions can also affect cognitive processes such as judgments and decisions (e.g., Bodenhausen et al., 1994) as well as memory performance (Miller and Leshner, 2007).

Overall, previous research showed that characteristics of news stories can affect both cognitive processes (e.g., judgments, decisions, and opinions) and diverse emotional responses (e.g., sadness, fear, and happiness), whereby studies on emotional responses appear to be underrepresented. We hence want to stress two critical aspects:

First, not only cognitive but also emotional responses to news stories may determine behavioral consequences in the long run. For example, news stories about a civil war in a foreign country may affect recipients' judgments about the safety of tourists and hence the willingness to visit this country, but sadness about many innocent victims of this war may also motivate donation behavior. Thus, we propose to pay attention to these behavioral consequences implied by the framing of news stories. In fact, news framing is a strong tool to significantly influence recipients' behavior by means of emotional processes. Therefore, the significance of specific news characteristics can perhaps be understood best by focusing on short-term emotional (and cognitive) responses as well as their implications for long-term behavioral consequences. In general, it is important to note that a news frame is “a central organizing idea or story line that provides meaning to an unfolding strip of events, weaving a connection among them. The frame suggests what the controversy is about, the essence of the issue” (Gamson and Modigliani, 1987, p.143). Thus, the framing of a news story is not only an esthetic issue but rather a regulating factor. In this sense, studies on the emotional responses to different news frames should be of broad social interest.

Second, the effects of emotional responses to news stories are diverse and previous research covered a wide range of negative and positive emotions. Knobloch-Westerwick and Keplinger (2007) proposed to consider suspense as one central emotional response and assumed that suspense positively influences the enjoyment of a news story indicated by one's reading appreciation and lingering interest in the news story. This idea is noteworthy as it bridges the conceptual gap between news and entertainment research: Zillmann (1996) outlined in his affective-disposition theory the central determinants of the enjoyment of the narrative in entertainment media. According to this theory, the experience of suspense is the striving force of immersion in and the enjoyment of fictional dramas, specified by the following preconditions: first, drama has to preoccupy itself with negative outcomes. Second, “liked protagonists who are deserving of good fortunes must be selected as targets for negative outcomes in order to make these outcomes feared and dreaded by the respondents” while, third, “high degrees of subjective certainty (rather than uncertainty) about the occurrence of outcomes that threaten liked protagonists must be created in the respondents” (p. 201). If these criteria are met, the intensity of experienced suspense should increase and hence positively affect the enjoyment of the narrative.

## The present studies

Assuming strong parallels between entertainment effects and news reception, Knobloch-Westerwick and Keplinger (2007; abbreviated by KWK) empirically tested whether Zillmann's model is also valid in the context of news stories. Hence, they were searching for counterevidence for the predominant distinction between entertainment processes and news reception processes. The present studies will pick up the findings reported by KWK, aiming to examine their replicability and to significantly expand them.

KWK applied the preconditions formulated by Zillmann (1996) to news consumption (instead of drama) and derived the following hypotheses:

**Hypothesis H1a:** *The intensity of experienced suspense during news exposure increases with the magnitude of the respondents' positive affective disposition toward protagonists in the news*.
**Hypothesis H1b:** *The intensity of experienced suspense during news exposure increases with the respondents' subjective certainty that the threatened harm will materialize, short of certainty about this outcome*.

Moreover, KWK addressed the observation that suspense attracts recipients to entertainment (Vorderer et al., 1996), leading to the assumption that suspense might also increase the liking of news:

**Hypothesis H1c:** *The liking of a news report (reading appreciation) increases with the level of suspense experienced during exposure to the report*.

Finally, as suspense should sustain until the outcome of a story is presented (Vorderer et al., 1996), it should motivate recipients to attend to continued news coverage as long as the outcome is unclear. Thus, in addition to the retrospectively rated reading appreciation, KWK examined in a prospective manner recipients' lingering interest in the news story:

**Hypothesis H1d:** *The motivation to attend to continued coverage of the topic increases with the level of suspense experienced during exposure to the report, given that no final resolution was provided*.

Overall, KWK found evidence for their hypotheses. More specifically, a higher likeability of the protagonist (H1a) and a likely negative outcome (H1b) increased the experienced suspense. Furthermore, reading appreciation (H1c) and lingering interest in the news story (H1d) positively correlated with the intensity of experienced suspense. Please note that we use a different numbering here (H1a–H1d) in contrast to KWK (H1–H4) for the sake of readability. Moreover, H1a and H1b refer to the effect of *manipulated* news characteristics (difference hypotheses), whereas H1c and H1d refer to the correlation between suspense experience and the subjective enjoyment of the news (correlation hypotheses).

Stimulated by the theoretical relevance of KWK's findings for news research and motivated by the emerging awareness of the need of replication studies in psychology and communication science (e.g., Bohannon, 2015; Vermeulen and Hartmann, 2015), we pursued to retest these original hypotheses. However, for the sake of generalizability, we created different news stories that also fulfilled the preconditions formulated by Zillmann (1996). Indeed, one may question that suspense does not act in the same way across various types of news. KWK examined the role of suspense in the context of two news stories that were comparable with stories usually met in entertainment contexts such as movies: illness caused by an unknown virus and kidnapping of the protagonist by a group of guerillas. It is conceivable that such types of news have an inherent potential to elicit suspense experiences (present Study 1) in contrast to less “spectacular” events or issues, such as political debates about affordable housing or fair working conditions for employees (Study 2) as well as the public transport system or compulsory vaccination (Study 3). Also, in some cases, the critical preconditions formulated by Zillmann are not met by the characteristics of an event. For example, sometimes it may be difficult to identify a unique protagonist, restricting the validity of Zillmann's model in general. Nonetheless, a large number of events do meet these requirements and hence make suspense a critical factor in the context of news stories.

In addition to the empirical support of their focal hypotheses, KWK found that a high vs. low likeability of the protagonist increased participants' reading appreciation as well as lingering interest in the news story. In contrast, the manipulated likelihood of a good/bad ending of the story had no significant effect on these two measurements. Importantly, the logic of KWK's arguments implies a mediation process: the likeability of the protagonist should intensify suspense which, in turn, should positively influence reading appreciation and lingering interest. KWK did not test this mediation hypothesis and hence it is unclear whether suspense actually acts as a mediator. Therefore, we expanded the set of hypotheses by explicitly addressing the mediation process. However, we did not ask whether suspense would mediate the effect of the *manipulated* likeability of the protagonist (dichotomous variable) on reading appreciation and lingering interest. Instead, we focused on participants' *reported* liking of the protagonist (continuous variable) because, on the one hand, the mediation hypothesis basically states a correlational relationship between subjective ratings and, on the other hand, to prevent a loss of information in the analysis. Indeed, this differentiation is important as manipulated news characteristics (e.g., the likeability of a news story's protagonist) do not necessarily equal the subjective impression of the recipients (e.g., their liking of the protagonist). This conceptual difference between manipulated and perceived characteristics of media content is an important issue not only limited to news (cf. Hamborg et al., 2014). Further, although not supported by the effects observed by KWK, we asked whether suspense would also mediate a potential correlation between the perceived likelihood of a good ending of the news story and reading appreciation/lingering interest. The different mediation hypotheses are subsumed under Hypothesis 2:

**Hypothesis H2a:** *The expected correlation between participants' liking of the protagonist of a news story and reading appreciation is mediated by the intensity of experienced suspense*.
**Hypothesis H2b:** *The expected correlation between participants' liking of the protagonist and lingering interest in the news story is mediated by the intensity of experienced suspense*.
**Hypothesis H2c:** *The expected correlation between the perceived likelihood of a good ending of a news story and reading appreciation is mediated by the intensity of experienced suspense*.
**Hypothesis H2d:** *The expected correlation between the perceived likelihood of a good ending and lingering interest in the news story is mediated by the intensity of experienced suspense*.

In addition to these hypotheses tested by all three of the present studies, Study 3 examined an empirically neglected but conceptually relevant aspect mentioned by KWK: while a likely negative outcome of a news story may increase recipients' reading appreciation when the protagonist and his personal fate has no relation to the recipients' own life, things may change when the news story becomes more personally relevant for the recipients. In the latter case, it might be that readers' liking of a news story benefits from a positive forecast because humans often show a preference for happy endings (Ross and Simonson, 1991). Moreover, Aust and Zillmann (1996) found that the distress potential of a news story and the perceived personal relevance were positively related. We hence formulated the following moderation hypothesis:

**Hypothesis H3:** *A high (vs. low) manipulated likelihood of a good ending increases participants' reading appreciation only when the news story is of high (vs. low) personal relevance*.

Finally, given that KWK found a positive correlation between experienced suspense and reading appreciation (H1c), we investigated in an exploratory manner whether the height of this correlation significantly changes when the news story is of higher personal relevance, that is, when the protagonist's personal fate is more closely linked to the recipient's own life. On the one hand, one might speculate that this correlation increases in the case of more personally relevant news when assuming that suspense acts as a general (positively connoted) pushing factor for the enjoyment of narratives. On the other hand, the correlation may decrease or its sign may even reverse because suspense may elicit threat when elicited by personally relevant stories. Hence, the answer to this question is of importance as it will indicate whether the experience of suspense is characterized by a universally positive valence or whether suspense can also show undesirable effects, challenging the suitability of suspense as a stylistic device in the context of news creation. Thus, we asked in an exploratory manner:

**Research Question RQ1:** *Does the positive correlation between experienced suspense and reading appreciation, which we expect when the personal relevance of the news story is low, significantly change when the news story becomes more personally relevant?*

## Study 1

Study 1 tested the original hypotheses of KWK (H1a–H1d) and the additional mediation hypotheses (H2a–H2d).

### Ethics statement

All procedures were performed in full accordance with the ethical guidelines of the German Psychological Society (DGPs, https://www.dgps.de/index.php?id=85). Also, if research objectives do not refer to issues regulated by law such as projects in the realm of medical and pharmacological research, which was the case in the present studies on news evaluation, no special permission by an ethics committee is required for psychological research in Germany. At the beginning of the study, participants were informed that the data of this study will be used for research purposes only and that all data are collected anonymously. Thus, no identifying information was collected and completion of the survey was considered to indicate consent. Participants who prematurely stopped the survey were not included in the analyses and all of their data were deleted from the dataset.

### Methods

#### Participants

All participants were enrolled in the study voluntarily. The link to the study was distributed via the mailing lists of several German universities and the social network Facebook. Participants were randomly assigned to Study 1 or Study 2 after clicking on the link. As KWK tested 272 participants in their study, this was our targeted sample size for Study 1. Finally, the data of 263 participants (210 females, *M*_*age*_ = 24.50, *SD*_*age*_ = 6.04) were analyzed (for exclusion criteria, see the Results Section). Overall, 126 participants had a university degree, 123 participants had a high school diploma, eight participants reported an advanced college certificate, and six participants reported a lower level of education. The majority of participants were students (*n* = 232), followed by employees (16), trainees (8), and others (7).

#### Study design and procedure

Following KWK, we conducted a computerized online experiment. Participants were randomly assigned to one of the four experimental conditions defined by the between-subject factors “manipulated likeability of the protagonist” (high vs. low) and “manipulated likelihood of a good ending” (high vs. low). Participants were initially introduced to the online study by a short study description. They were told to read and evaluate two news stories of a current newspaper. Afterwards, they provided demographic data (sex, age, highest educational achievement, current job position). Then, they were presented two fictional news stories in random order, serving as the within-subject factor “news story” (accident vs. kidnapping). KWK also employed two news stories “to gain more reliable data from two measurements” (p. 199). However, as we observed rather low internal reliabilities across measurements, we introduced the news stories as a within-subject factor, allowing a direct test of effect generalizability. The selection of news topics followed the study of KWK in favor of our initial replication attempt. As outlined above, these news stories were characterized by events that may have an inherent potential to elicit suspense. Following KWK, the two news stories were of similar length. The original texts and English translations are presented in full length in an online Supplementary File (S1). One of the two stories read as follows:

After a car accident on a rural road close to Ismaning, Munich, Maria Nuckertid (34) was brought to hospital. *She gained fame for her charity organization “All children are equal” which gives financial support to families with disabled children*. Eye witnesses reported that her car went off the street due to flash freeze and then frontally bumped into a tree. One witness directly called the ambulance, pulled the unconscious person out of the car, and gave first aid until the ambulance arrived. Maria Nuckertid was treated by the emergency doctor at the accident location and later on operated in hospital. The attending doctor of the trauma surgery ward declared that she suffered from a craniocerebral injury and is still unconscious. *Several examinations revealed that her condition is stable and not life-threatening anymore. As per her doctor, there is a high probability that she will awake from coma very soon*. At the moment, Maria Nuckertid is in intensive care being shepherded by her partner. Further examinations and updates will follow in the next days, the speaker of the hospital assured.

This scenario was intended to elicit a positive affective disposition toward the protagonist and the impression of a high likelihood of a good ending. In the news scenario that was intended to elicit a negative affective disposition toward the protagonist it said: “*She gained fame for a mistreatment and squalidness of her three children and a resulting fight with the youth welfare service*.” In the news scenario that was intended to elicit the impression of a low likelihood of a good ending it said: “*Several examinations revealed that her condition is still unstable and life-threatening. As per her doctor, there is a high probability that she will not awake from coma*.”

Each news story was followed by a set of evaluation questions (dependent variables). At the end of the study, participants were thanked for their participation and were debriefed about the fictitious nature of the presented news stories.

#### Dependent variables

The participants evaluated the two news stories on several 5-point items ranging from one (*not at all*) to five (*very*). Participants rated their liking of the protagonist (*How likable was [protagonist's name] to you?* English translation) and the likelihood of a good ending (*How likely do you think is it that [protagonist's name] will [good ending]?*). Note that a higher rating on the second scale indicates a higher likelihood of a good ending. We will present the results according to this scale to facilitate the understanding of the results, in contrast to KWK who presented their results with an emphasis on a bad ending.

We used three items to assess the experienced suspense (*How much do the following characteristics apply to the text?*): gripping, thrilling, exciting. Following Knobloch et al. (2004), these items reliably showed the highest loadings on a suspense scale across several studies and a good coefficient alpha reliability of 0.86 and 0.88 for the two news stories in the present study. Following KWK, participants' liking of a news story (reading appreciation) was measured by the question “*How did you like the story in general?;*” participants lingering interest in the news story was measured by the question “*How interested are you to keep yourself informed during the next days on what is happening to [protagonist's name]?*.”

Additionally, the perceived personal relevance of a news story was measured by the following item: “*How much does this story concern you and your life*?” The participants were also asked “*How informative was this news for you*?” in order to check whether a higher personal relevance of a news story was accompanied by a higher perceived utility of information, as questioned by KWK.

### Results and discussion

First of all, we excluded 32 participants prior to the analysis that was finally based on 263 participants. Following previous studies on news evaluation (Kaspar et al., 2015a,c), we applied a two-step procedure to exclude participants who were either too fast or too slow, which may indicate a lack of engagement or distracted attention, a common concern in online studies. Firstly, participants were excluded when their completion time was part of the lower or upper 5% of the sample. Secondly, we calculated the mean completion time and excluded, in accordance with Miller (1991) and Selst and Jolicoeur (1994), participants with a completion time that deviated more than three standard deviations from the mean.

#### Manipulation checks

As intended, the reported liking of the protagonist was higher in the group that read the text version presenting a highly (vs. less) likable protagonist. This effect was found for the “accident story” (*M* = 3.57, *SD* = 0.77 vs. *M* = 1.94, *SD* = 0.91), *t*_(261)_ = 15.568, *p* < 0.001, *d* = 1.926, and for the “kidnapping story” (*M* = 3.65, *SD* = 0.74 vs. *M* = 2.12, *SD* = 0.91), *t*_(258.360)_ = 14.973, *p* < 0.001, *d* = 1.827.

Also, the perceived likelihood of a good ending of the news story was higher when the participants read the text version that was intended to elicit the impression of a likely good (vs. bad) outcome. This effect was significant for the “accident story” (*M* = 3.81, *SD* = 0.80 vs. *M* = 2.39, *SD* = 0.86), *t*_(261)_ = 13.878, *p* < 0.001, *d* = 1.714, and for the “kidnapping story” (*M* = 3.77, *SD* = 0.68 vs. *M* = 2.80, *SD* = 0.84), *t*_(238.162)_ = 10.236, *p* < 0.001, *d* = 1.279. Thus, the manipulations were successful in both news stories.

Finally, the reported personal relevance of the two news stories was *M* = 1.70 (*SD* = 0.74) and the utility of information was *M* = 3.27 (*SD* = 0.87).

#### Do a high likeability of the protagonist (H1a) and a likely bad outcome (H1b) increase perceived suspense?

We calculated a 2 (manipulated likeability of the protagonist) × 2 (manipulated likelihood of a good ending) × 2 (news story) mixed-measures ANOVA with experienced suspense as dependent variable.

**Figure 1:**
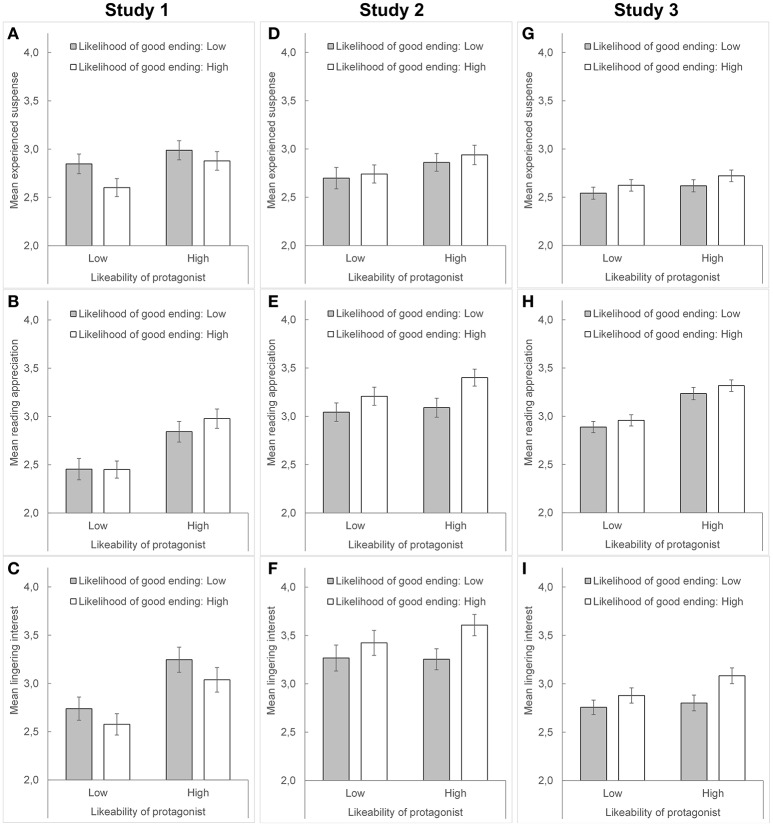
**The main effects of the manipulated likelihood of a good ending (low vs. high) and the manipulated likeability of the protagonists (low vs. high) found in Studies 1, 2, and 3 (means across two different news stories)**. The dependent variables are the experienced suspense during reading **(A,D, and G)**, reading appreciation **(B,E, and H)**, and lingering interest in the news story **(C,F, and I)**.

Supporting H1a, a high vs. low (manipulated) likeability of the protagonist increased the experienced suspense (high liking: *M* = 2.93, *SD* = 0.77; low liking: *M* = 2.72, *SD* = 0.82), *F*_(1, 259)_ = 4.518, *p* = 0.034, $\eta_{p}^{2}$ = 0.017 (see Figure 1A). The manipulated likeability of the protagonist did not interact with any other factor, all *F*s ≤ 2.084, *p*s ≥ 0.150, $\eta_{p}^{2}$ ≤ 0.008.

In partial support of H1b, as shown in Figure 1A, a high vs. low (manipulated) likelihood of a good ending decreased, by trend, the experienced suspense (high likelihood: *M* = 2.74, *SD* = 0.80; low likelihood: *M* = 2.91, *SD* = 0.80), *F*_(1, 259)_ = 3.272, *p* = 0.072, $\eta_{p}^{2}$ = 0.012. The manipulated likelihood of a good ending did not interact with any other factor, all *F*s ≤ 0.818, *p*s ≥ 0.366, $\eta_{p}^{2}$ ≤ 0.003.

Hence, although we found a pronounced main effect of the news story on the experienced suspense, *F*_(1, 259)_ = 75.760, *p* < 0.001, $\eta_{p}^{2}$ = 0.226, with higher suspense in the “kidnapping story” (*M* = 3.07, *SD* = 0.93) compared to the “accident story” (*M* = 2.57, *SD* = 0.92), the news story did not moderate the exclusive main effects of the other two factors on experienced suspense.

#### Do reading appreciation (H1c) and lingering interest (H1d) positively correlate with experienced suspense?

Supporting H1c, the intensity of experienced suspense positively correlated with reading appreciation in both the “accident story,” *r* = 0.344, *p* < 0.001, and the “kidnapping story,” *r* = 0.317, *p* < 0.001. Based on Fisher's *r*-to-*z* transformation, we found that the two correlation coefficients did not differ, *z* = 0.35, *p* = 0.726.

Supporting H1d, the intensity of experienced suspense also positively correlated with the extent of lingering interest in both the “accident story,” *r* = 0.472, *p* < 0.001, and the “kidnapping story,” *r* = 0.561, *p* < 0.001. The two correlation coefficients did not significantly differ, *z* = 1.39, *p* = 0.165.

To conclude, all original hypotheses were supported by the present data, replicating the result pattern reported by KWK.

#### Is the expected correlation between participants' liking of the protagonist and reading appreciation (H2a)/lingering interest (H2b) mediated by the intensity of experienced suspense?

We tested the mediation hypotheses by using the PROCESS macro (Model 4) of Hayes (2016) that allows testing indirect effects by means of a bootstrapping procedure (50,000 bootstrapped samples). With respect to H2a, as shown in Table 1, besides a positive effect of the reported liking of the protagonist on suspense, we found a positive total and a positive direct effect of the reported liking of the protagonist on reading appreciation. Further, and in accordance with H2a, the analysis of the indirect effect revealed that this relationship was additionally (i.e., partially) mediated by the experienced suspense as zero was not included in the bootstrapped 99% confidence interval. The type of news story did not affect the overall result pattern. Regarding H2b, we found evidence for a positive total and direct effect as well as for a mediation process. These effects were found for both news stories (see Table 1).

**Table 1:** **Analyses of indirect effects for mediation models of Study 1 described in H2a and H2b**.

**Pathway**	**Accident story**				**Kidnapping story**			
	***Coeff***.	***SE***	***t***	***p***	***Coeff***.	***SE***	***t***	***p***
**H2a**								
Model summary	*R^2^* = 0.174, *F*_(2, 260)_ = 27.357, *p* < 0.001^***^				*R^2^* = 0.139, *F*_(2, 260)_ = 21.026, *p* < 0.001^***^			
Effect of IV on mediator	0.191	0.047	4.043	<0.001^***^	0.206	0.050	4.155	<0.001^***^
Direct effect of mediator on DV	0.306	0.062	4.907	<0.001^***^	0.278	0.062	4.490	<0.001^***^
Total effect of IV on DV	0.264	0.050	5.305	<0.001^***^	0.232	0.051	4.516	<0.001^***^
Direct effect of IV on DV	0.205	0.049	4.178	<0.001^***^	0.175	0.051	3.413	0.001^***^
Indirect effect of IV on DV through mediator	effect = 0.059 (*SE* = 0.020), 99%*CI*: 0.017–0.124^**^				effect = 0.057 (*SE* = 0.022), 99%*CI*: 0.014–0.128^**^			
**H2b**								
Model summary	*R^2^* = 0.268, *F*_(2, 260)_ = 47.635, *p* < 0.001^***^				*R^2^* = 0.345, *F*_(2, 260)_ = 68.584, *p* < 0.001^***^			
Effect of IV on mediator	0.191	0.047	4.043	<0.001^***^	0.206	0.050	4.155	<0.001^***^
Direct effect of mediator on DV	0.563	0.074	7.655	<0.001^***^	0.639	0.064	9.968	<0.001^***^
Total effect of IV on DV	0.340	0.062	5.482	<0.001^***^	0.315	0.060	5.240	<0.001^***^
Direct effect of IV on DV	0.232	0.058	4.017	<0.001^***^	0.184	0.053	3.472	0.001^***^
Indirect effect of IV on DV through mediator	effect = 0.108 (*SE* = 0.029), 99%*CI*: 0.040–0.193^**^				effect = 0.131 (*SE* = 0.034), 99%*CI*: 0.051–0.224^**^			

*H2a: IV = liking of protagonist, Mediator = experienced suspense, DV = reading appreciation*.

*H2b: IV = liking of protagonist, Mediator = experienced suspense, DV = lingering interest*.

** *p < 0.01*,

*** *p < 0.001*.

*The table depicts the model summaries, unstandardized coefficients, and the point estimate for the indirect effects*.

#### Is the expected correlation between the perceived likelihood of a good ending of a news story and reading appreciation (H2c)/lingering interest (H2d) mediated by the intensity of experienced suspense?

With respect to H2c, as shown in Table 2, we only found the already observed positive correlations between the mediator and the outcome variable (see above). No further regression coefficient reached statistical significance. Also, there was no hint for mediation processes. The same result pattern was found regarding H2d. Hence, when we substituted the independent variable of the mediation model, all effects disappeared.

**Table 2:** **Analyses of indirect effects for mediation models of Study 1 described in H2c and H2d**.

**Pathway**	**Accident story**				**Kidnapping story**			
	***Coeff***.	***SE***	***t***	***p***	***Coeff***.	***SE***	***t***	***p***
**H2c**								
Model summary	*R^2^* = 0.119, *F*_(2, 260)_ = 17.587, *p* < 0.001^***^				*R^2^* = 0.102, *F*_(2, 260)_ = 14.716, *p* < 0.001^***^			
Effect of IV on mediator	−0.081	0.052	−1.563	0.119	−0.075	0.064	−1.180	0.239
Direct effect of mediator on DV	0.373	0.063	5.930	< 0.001^***^	0.333	0.062	5.423	< 0.001^***^
Total effect of IV on DV	−0.005	0.056	−0.089	0.929	0.010	0.067	0.148	0.883
Direct effect of IV on DV	0.025	0.053	0.477	0.634	0.035	0.064	0.550	0.583
Indirect effect of IV on DV through mediator	effect = −0.030 (*SE* = 0.021), 90%*CI*: −0.068–0.001				effect = −0.025 (*SE* = 0.025), 90%*CI*: −0.072–0.012			
**H2d**								
Model summary	*R^2^* = 0.224, *F*_(2, 260)_ = 37.551, *p* < 0.001^***^				*R^2^* = 0.316, *F*_(2, 260)_ = 60.057, *p* < 0.001^***^			
Effect of IV on mediator	−0.081	0.052	−1.563	0.119	−0.075	0.064	−1.180	0.239
Direct effect of mediator on DV	0.630	0.074	8.534	< 0.001^***^	0.691	0.064	10.869	< 0.001^***^
Total effect of IV on DV	−0.093	0.070	−1.333	0.184	−0.093	0.079	−1.170	0.243
Direct effect of IV on DV	−0.042	0.062	−0.677	0.499	−0.040	0.066	−0.613	0.541
Indirect effect of IV on DV through mediator	effect = −0.051 (*SE* = 0.035), 90%*CI*: −0.111–0.004				effect = −0.052 (*SE* = 0.051), 90%*CI*: −0.138–0.028			

*H2c: IV = perceived likelihood of good ending, Mediator = experienced suspense, DV = reading appreciation*.

*H2d: IV = perceived likelihood of good ending, Mediator = experienced suspense, DV = lingering interest*.

*** *p < 0.001*.

*The table depicts the model summaries, unstandardized coefficients, and the point estimate for the indirect effects*.

#### Effects of manipulated news characteristics on reading appreciation and lingering interest

With respect to reading appreciation, the 2 × 2 × 2 ANOVA revealed a main effect of the news story, *F*_(1, 259)_ = 30.011, *p* < 0.001, $\eta_{p}^{2}$ = 0.104, and a main effect of the manipulated likeability of the protagonist, *F*_(1, 259)_ = 19.937, *p* < 0.001, $\eta_{p}^{2}$ = 0.071. The two factors also interacted, *F*_(1, 259)_ = 4.002, *p* = 0.046, $\eta_{p}^{2}$ = 0.015. Supporting the observation by KWK, a high vs. low (manipulated) likeability of the protagonist increased the reading appreciation (see Figure 1B), whereby this increase was larger in the “accident story” (*M* = 2.81, *SD* = 0.98 vs. *M* = 2.23, *SD* = 0.93, *t* = 4.906, *p* < 0.001) compared to the “kidnapping story” (*M* = 3.02, *SD* = 0.92 vs. *M* = 2.68, *SD* = 0.99, *t* = 2.955, *p* = 0.003). No further effects were found, all *F*s ≤ 2.245, *p*s ≥ 0.135, $\eta_{p}^{2}$ ≤ 0.009.

In complete accordance with KWK's observation, lingering interest in the news story was increased by a high vs. low (manipulated) likeability of the protagonist (*M* = 3.13, *SD* = 1.02 vs. *M* = 2.66, *SD* = 0.96), *F*_(1, 259)_ = 15.723, *p* < 0.001, $\eta_{p}^{2}$ = 0.057 (see Figure 1C). Moreover, the “kidnapping story” (*M* = 3.24, *SD* = 1.15) elicited a higher reading appreciation compared to the “accident story” (*M* = 2.52, *SD* = 1.24), *F*_(1, 259)_ = 82.093, *p* < 0.001, $\eta_{p}^{2}$ = 0.241. Besides these two main effects, no further significant effects were found, all *F*s ≤ 2.307, *p*s ≥ 0.130, $\eta_{p}^{2}$ ≤ 0.009.

To conclude, while the manipulated likeability of the protagonist affected both reading appreciation and lingering interest, the manipulated likelihood of a good ending did not affect these two measurements.

### Summary

The manipulations of the protagonist's likeability and of the likelihood of a good ending were successful in both news stories. A high vs. low (manipulated) likeability of the protagonist increased the experienced suspense, supporting H1a. A high vs. low (manipulated) likelihood of a good ending decreased, by trend, the experienced suspense, partially supporting H1b. Reading appreciation and lingering interest positively correlated with experienced suspense, supporting H1c and H1d, respectively. Also, a high vs. low (manipulated) likeability of the protagonist increased reading appreciation and lingering interest. All these effects were observed for both news stories, indicating some generalizability and a perfect replication of the result pattern reported by KWK.

Moreover, supporting H2a/H2b, we found a positive correlation between the reported liking of the protagonist and reading appreciation/lingering interest. This relationship was partially mediated by experienced suspense and it was independent of the news story. In contrast, contradicting H2c/H2d, no correlation was found between the likelihood of a good ending and reading appreciation/lingering interest. There was also no hint for an indirect effect in the absence of a total or direct effect.

To conclude, suspense was not only affected by the manipulated characteristics of the news stories (i.e., the protagonist's likeability and the likelihood of a good ending). Suspense also significantly mediated the relationship between, on the one hand, participants' actual perception of the news characteristics (i.e., the reported liking of the protagonist and the perceived likelihood of a good ending) and, on the other hand, reading appreciation and lingering interest in the news story. Hence, suspense appears to be a critical emotional response mediating news evaluation and motivational tendencies. Thereby, the result pattern is compatible with the predictions derived from Zillmann's (1996) affective-disposition theory, indicating that the theory is not only valid in the context of entertainment media but also in the context of news stories.

## Study 2

Study 1 showed that the effects found by KWK were replicable a decade later with a similar set of news stories. Study 2 aimed to provide further evidence for the generalizability of the original results. We used a new set of news stories whose personal relevance for the recipients was significantly higher than in Study 1, while the preconditions for suspense formulated by Zillmann (1996) have been met again. Moreover, in contrast to the news stories used in Study 1, the news stories of Study 2 can be considered as less “spectacular” political debates about affordable housing or fair working conditions for employees. These two topics were considered as timely and relevant for our target population of young adults. The critical question was whether suspense nonetheless remains an important variable in this context.

### Method

The data of 255 participants (200 females, *M*_*age*_ = 24.26, *SD*_*age*_ = 5.71) were analyzed. Overall, 115 participants had a university degree, 118 participants had a high school diploma, 12 participants reported an advanced college certificate, and 10 participants reported a lower level of education. Again, the majority of participants were students (*n* = 216), followed by employees (28), trainees (7), and others (4). All participants did not participate in Study 1 as the survey software randomly assigned them to either Study 1 or Study 2.

The study design, procedure, and measurements were identical to those used in Study 1. The three items that measured suspense showed a good coefficient alpha reliability of 0.81 for the two news stories “fair working conditions” and “affordable housing” (for the news stories, see the online Supplementary File S1).

### Results and discussion

Prior to the analyses, we excluded 36 participants according to the procedure applied in Study 1.

#### Manipulation checks

As intended, the reported liking of the protagonist was higher in the group that read the text version presenting a highly (vs. less) likable protagonist. This effect was found for the “fair working conditions story” (*M* = 3.58, *SD* = 0.78 vs. *M* = 3.00, *SD* = 0.68), *t*_(251.989)_ = 6.265, *p* < 0.001, *d* = 0.781, and for the “affordable housing story” (*M* = 3.54, *SD* = 0.76 vs. *M* = 3.13, *SD* = 0.83), *t*_(253)_ = 4.108, *p* < 0.001, *d* = 0.515.

The perceived likelihood of a good ending of the news story was higher when the participants read the text version that was intended to elicit the impression of a likely good (vs. bad) outcome. This effect was significant for the “fair working conditions story” (*M* = 2.94, *SD* = 1.00 vs. *M* = 2.37, *SD* = 0.91), *t*_(253)_ = 4.750, *p* < 0.001, *d* = 0.595, and for the “affordable housing story” (*M* = 3.04, *SD* = 0.97 vs. *M* = 2.19, *SD* = 0.84), *t*_(250.386)_ = 7.469, *p* < 0.001, *d* = 0.933.

Additionally, we compared the reported personal relevance of the news story between Studies 1 and 2. As intended, the two news stories of Study 2 (“fair working conditions story:” *M* = 2.67, *SD* = 1.15; “affordable housing story:” *M* = 3.60, *SD* = 1.19) were perceived as more personally relevant than the two news stories presented in Study 1 (“accident story:” *M* = 1.72, *SD* = 1.00; “kidnapping story:” *M* = 1.68, *SD* = 0.82), all *t*s ≥ 10.033, *p*s < 0.001, *d*s ≥ 0.884. Thereby, a higher personal relevance of the news story was not necessarily accompanied by a higher perceived utility of information. The participants rated the “accident story” of Study 1 (low personal relevance) as less informative than the “affordable housing story” of Study 2 (high personal relevance), but they also rated the “kidnapping story” of Study 1 as more informative than the “fair working conditions story” of Study 2, both |*t*s| ≥ 3.155, *p*s ≤ 0.002, *d*s ≥ 0.277. The other two contrasts did not reach statistical significance, both |*t*s| ≤ 1.487, *p*s ≥ 0.138, *d*s ≤ 0.131.

#### Do a high likeability of the protagonist (H1a) and a likely bad outcome (H1b) increase perceived suspense?

We again calculated the 2 (manipulated likeability of the protagonist) × 2 (manipulated likelihood of a good ending) × 2 (news story) mixed-measures ANOVA with experienced suspense as dependent variable.

In partial support of H1a, a high vs. low (manipulated) likeability of the protagonist increased, by trend, the experienced suspense (high: *M* = 2.90, *SD* = 0.78; low: *M* = 2.72, *SD* = 0.80), *F*_(1, 251)_ = 3.310, *p* = 0.070, $\eta_{p}^{2}$ = 0.013 (see Figure 1D).

Contrary to H1b, suspense was not affected by the manipulated likelihood of a good ending, *F*_(1, 251)_ = 0.371, *p* = 0.543, $\eta_{p}^{2}$ = 0.001, but a main effect of the news story occurred, *F*_(1, 251)_ = 16.265, *p* < 0.001, $\eta_{p}^{2}$ = 0.061, because the “affordable housing story” (*M* = 2.91, *SD* = 0.90) elicited more suspense than the “fair working conditions story” (*M* = 2.71, *SD* = 0.87). No further effects were found, all *F*s ≤ 0.186, *p*s ≥ 0.667, $\eta_{p}^{2}$ ≤ 0.001.

#### Do reading appreciation (H1c) and lingering interest (H1d) positively correlate with experienced suspense?

Supporting H1c, the intensity of experienced suspense positively correlated with the reading appreciation in both the “fair working conditions story,” *r* = 0.355, *p* < 0.001, and the “affordable housing story,” *r* = 0.502, *p* < 0.001. However, the latter correlation was significantly higher than the former, *z* = 2.03, *p* = 0.042, indicating a moderating role of the news story. This result differed from those found in Study 1 and shed some critical light on the generalizability of effects across news stories in absolute (but not relative) terms.

Supporting H1d, the intensity of experienced suspense also positively correlated with the extent of lingering interest in both the “fair working conditions story,” *r* = 0.580, *p* < 0.001, and the “affordable housing story,” *r* = 0.564, *p* < 0.001. These two correlation coefficients did not differ, *z* = 0.27, *p* = 0.787.

Overall, we were able to replicate most of the effects found in Study 1 and by KWK, except the missing influence of the manipulated likelihood of a good ending on suspense.

#### Is the expected correlation between participants' liking of the protagonist and reading appreciation (H2a)/lingering interest (H2b) mediated by the intensity of experienced suspense?

The test of the mediation hypotheses revealed the result pattern found in Study 1, as shown in Table 3. In short, independent of the type of news story, we found a positive total and a positive direct effect of the reported liking of the protagonist on both reading appreciation and lingering interest. These correlations were additionally (i.e., partially) mediated by experienced suspense, supporting H2a and H2b.

**Table 3:** **Analyses of indirect effects for mediation models of Study 2 described in H2a and H2b**.

**Pathway**	**Fair working conditions story**				**Affordable housing story**			
	***Coeff***.	***SE***	***t***	***p***	***Coeff***.	***SE***	***t***	***p***
**H2a**								
Model summary	*R^2^* = 0.177, *F*_(2, 252)_ = 27.022, *p* < 0.001^***^				*R^2^* = 0.287, *F*_(2, 252)_ = 50.647, *p* < 0.001^***^				
Effect of IV on mediator	0.445	0.063	7.038	<0.001^***^	0.515	0.061	8.439	<0.001^***^
Direct effect of mediator on DV	0.260	0.064	4.090	<0.001^***^	0.403	0.060	6.677	<0.001^***^
Total effect of IV on DV	0.391	0.066	5.927	<0.001^***^	0.441	0.063	6.956	<0.001^***^
Direct effect of IV on DV	0.275	0.070	3.931	<0.001^***^	0.233	0.066	3.524	0.001^***^
Indirect effect of IV on DV through mediator	effect = 0.116 (*SE* = 0.035), 99%*CI*: 0.040–0.219^**^				effect = 0.207 (*SE* = 0.044), 99%*CI*: 0.103–0.332^**^				
**H2b**								
Model summary	*R^2^* = 0.347, *F*_(2, 252)_ = 66.963, *p* < 0.001^***^				*R^2^* = 0.329, *F*_(2, 252)_ = 61.650, *p* < 0.001^***^				
Effect of IV on mediator	0.445	0.063	7.038	<0.001^***^	0.515	0.061	8.439	<0.001^***^
Direct effect of mediator on DV	0.737	0.077	9.615	<0.001^***^	0.638	0.073	8.715	<0.001^***^
Total effect of IV on DV	0.497	0.090	5.520	<0.001^***^	0.489	0.081	6.044	<0.001^***^
Direct effect of IV on DV	0.169	0.084	1.999	0.047^*^	0.160	0.080	1.994	0.047^*^
Indirect effect of IV on DV through mediator	effect = 0.328 (*SE* = 0.059), 99%*CI*: 0.194–0.495^**^				effect = 0.328 (*SE* = 0.052), 99%*CI*: 0.208–0.481^**^				

*H2a: IV = liking of protagonist, Mediator = experienced suspense, DV = reading appreciation*.

*H2b: IV = liking of protagonist, Mediator = experienced suspense, DV = lingering interest*.

* *p < 0.05*,

** *p < 0.01*,

*** *p < 0.001*.

*The table depicts the model summaries, unstandardized coefficients, and the point estimate for the indirect effects*.

#### Is the expected correlation between the perceived likelihood of a good ending of a news story and reading appreciation (H2c)/lingering interest (H2d) mediated by the intensity of experienced suspense?

With respect to H2c and H2d, the results were completely different compared to Study 1 where we did not find any total, direct, or indirect effects. As shown in Table 4, the perceived likelihood of a good ending of the news story showed a positive total and direct effect on reading appreciation. We also observed an indirect effect mediated by suspense, supporting H2c. These effects were relatively independent of the type of news story. Moreover, we found a positive total effect of the perceived likelihood of a good ending on lingering interest, but no direct effect. However, suspense (completely) mediated this relationship in both news stories. Importantly, the likelihood of a good ending was positively associated with experienced suspense, reading appreciation, and lingering interest. This result clearly contradicts the assumption that likely negative outcomes are beneficial for suspense experiences and the enjoyment of narratives (cf. Zillmann, 1996).

**Table 4:** **Analyses of indirect effects for mediation models of Study 2 described in H2c and H2d**.

**Pathway**	**Fair working conditions story**				**Affordable housing story**			
	***Coeff***.	***SE***	***t***	***p***	***Coeff***.	***SE***	***t***	***p***
**H2c**								
Model summary	*R^2^* = 0.170, *F*_(2, 252)_ = 25.824, *p* < 0.001^***^				*R^2^* = 0.260, *F*_(2, 252)_ = 44.270, *p* < 0.001^***^			
Effect of IV on mediator	0.192	0.053	3.601	<0.001^***^	0.214	0.055	3.911	<0.001^***^
Direct effect of mediator on DV	0.313	0.060	5.228	<0.001^***^	0.480	0.056	8.584	<0.001^***^
Total effect of IV on DV	0.250	0.053	4.693	<0.001^***^	0.188	0.055	3.398	0.001^***^
Direct effect of IV on DV	0.190	0.052	3.655	<0.001^***^	0.085	0.050	1.694	0.092^+^
Indirect effect of IV on DV through mediator	effect = 0.060 (*SE* = 0.021), 99%*CI*: 0.017–0.127^**^				effect = 0.103 (*SE* = 0.029), 99%*CI*: 0.037–0.187^**^			
**H2d**								
Model summary	*R^2^* = 0.343, *F*_(2, 252)_ = 65.654, *p* < 0.001^***^				*R^2^* = 0.323, *F*_(2, 252)_ = 60.122, *p* < 0.001^***^			
Effect of IV on mediator	0.192	0.053	3.601	<0.001^***^	0.214	0.055	3.911	<0.001^***^
Direct effect of mediator on DV	0.775	0.072	10.748	<0.001^***^	0.684	0.067	10.236	<0.001^***^
Total effect of IV on DV	0.243	0.074	3.298	0.001^***^	0.229	0.069	3.312	0.001^***^
Direct effect of IV on DV	0.094	0.063	1.503	0.134	0.082	0.060	1.375	0.171
Indirect effect of IV on DV through mediator	effect = 0.149 (*SE* = 0.043), 99%*CI*: 0.041–0.268^**^				effect = 0.147 (*SE* = 0.040), 99%*CI*: 0.051–0.254^**^			

*H2c: IV = perceived likelihood of good ending, Mediator = experienced suspense, DV = reading appreciation*.

*H2d: IV = perceived likelihood of good ending, Mediator = experienced suspense, DV = lingering interest*.

+ *p < 0.10*,

** *p < 0.01*,

*** *p < 0.001*.

*The table depicts the model summaries, unstandardized coefficients, and the point estimate for the indirect effects*.

#### Effects of manipulated news characteristics on reading appreciation and lingering interest

We computed the 2 × 2 × 2 ANOVA with reading appreciation as dependent variable. In contrast to Study 1, we found a main effect of the manipulated likelihood of a good ending, *F*_(1, 251)_ = 6.376, *p* = 0.012, $\eta_{p}^{2}$ = 0.025. As shown in Figure 1E, a high vs. low (manipulated) likelihood of a good ending increased the reading appreciation (high: *M* = 3.30, *SD* = 0.74; low: *M* = 3.07, *SD* = 0.77). We also found a main effect of the news story, *F*_(1, 251)_ = 34.018, *p* < 0.001, $\eta_{p}^{2}$ = 0.119, with higher ratings for the “affordable housing story” (*M* = 3.36, *SD* = 0.90) compared to the “fair working conditions story” (*M* = 3.02, *SD* = 0.88), but we did not observe further effects, all *F*s ≤ 1.611, *p*s ≥ 0.206, $\eta_{p}^{2}$ ≤ 0.006.

Finally, we computed the 2 × 2 × 2 ANOVA with lingering interest in the news story as dependent variable. In complete contrast to KWK and Study 1, participants' lingering interest was increased in the case of a high vs. low (manipulated) likelihood of a good ending (high likelihood: *M* = 3.52, *SD* = 0.97; low likelihood: *M* = 3.26, *SD* = 0.95), *F*_(1, 251)_ = 4.446, *p* = 0.036, $\eta_{p}^{2}$ = 0.017 (see Figure 1F), whereas it was not affected by the manipulated likeability of the protagonist, *F*_(1, 251)_ = 0.501, *p* = 0.480, $\eta_{p}^{2}$ = 0.002. An additional effect of the news story occurred, *F*_(1, 251)_ = 40.634, *p* < 0.001, $\eta_{p}^{2}$ = 0.139, whereby the “affordable housing story” (*M* = 3.65, *SD* = 1.12) elicited a stronger lingering interest than the “fair working conditions story” (*M* = 3.13, *SD* = 1.20). No further effects were found, all *F*s ≤ 0.671, *p*s ≥ 0.413, $\eta_{p}^{2}$ ≤ 0.003.

### Summary

The manipulations of the protagonist's likeability and of the likelihood of a good ending were successful in both news stories. In contrast to Study 1, the present news stories were rated as more personally relevant. This time, a high vs. low (manipulated) likeability of the protagonist increased the experienced suspense only by trend (H1a), while the manipulated likelihood of a good ending did not significantly affect suspense, contradicting H1b. Supporting H1c/H1d, reading appreciation and lingering interest positively correlated with experienced suspense. Again, these effects were found for both news stories.

In contrast to Study 1, we found no effect of the manipulated likeability of the protagonist on reading appreciation and lingering interest. Rather, a high vs. low (manipulated) likelihood of a good ending increased participants' reading appreciation and lingering interest, while these effects were not moderated by the type of news story. Hence, this result pattern completely differed from those observed in Study 1 and by KWK.

Moreover, this time we found a considerable discrepancy between effects related to manipulated vs. actually perceived news characteristics: the manipulated likelihood of a good ending did not affect suspense, but the perceived likelihood of a good ending positively correlated with suspense. Further, no effects of the protagonist's manipulated likeability on reading appreciation and lingering interest were found, but participants' reported liking of the protagonist positively correlated with both variables. Thus, the results of Study 2 demonstrate that correlations between perceived news characteristics and indicators of the enjoyment of news stories might exist even when the manipulation of news characteristics does not affect the enjoyment of news stories.

In accordance with Study 1, and supporting H2a/H2b, we found a positive correlation between the reported liking of the protagonist and reading appreciation/lingering interest that was partially mediated by experienced suspense in both news stories. In contrast to Study 1, we found support for H2c: the perceived likelihood of a good ending showed a total and a direct effect on reading appreciation that was additionally (i.e., partially) mediated by suspense. This time, we also found support for H2d as we observed a total effect of the perceived likelihood of a good ending on lingering interest, while suspense completely mediated this relationship. Again, these results were found for both news stories.

Overall, we found only partial support for the results previously observed. Importantly, and in strong contrast to Study 1 and KWK, the likelihood of a good ending was positively associated with experienced suspense, reading appreciation, and lingering interest. This result clearly contradicts the assumption that likely negative outcomes are beneficial for suspense experiences and the enjoyment of narratives, as stated by Zillmann (1996) in the context of his affective-disposition theory.

## Study 3

Study 2 aimed to provide further evidence for the effects found in Study 1 and by KWK. We found some considerable differences in the result patterns. Hence, we may speculate that the personal relevance of the news stories, which was higher in Study 2, accounts for these differences. However, the news of the two studies did not only differ in their personal relevance but also in many other uncontrolled variables that may explain some of the different results. We therefore conducted Study 3, in which we used a set of news stories that allowed the systematic variation of their personal relevance across experimental conditions to examine potential interactions between personal relevance and the other manipulated news characteristics (H3 and RQ1). Following Study 2, the two news stories again covered topics of broad social interest.

### Method

The data of 599 participants (445 females, *M*_*age*_ = 26.28, *SD*_*age*_ = 7.44) were analyzed (exclusion criteria as in Studies 1 and 2). Overall, 185 participants had a university degree, 358 participants had a high school diploma, 38 participants reported an advanced college certificate, and 18 participants reported a lower level of education. Again, the majority of participants were students (476), followed by employees (97), others (22), and trainees (4).

The study design, procedure, and measurements were identical to those applied in Studies 1 and 2. The three items measuring suspense showed a good coefficient alpha reliability of 0.79 for the two news stories “public transport” and “compulsory vaccination” (see the online Supplementary File S1). Following Knobloch et al. (2002), we manipulated the personal relevance of a news story by varying the region in which the story took place (high relevance: Germany; low relevance: Denmark/Norway).

### Results and discussion

#### Manipulation checks

As intended, the reported liking of the protagonist was higher in the group who read the text version presenting a highly (vs. less) likable protagonist. This effect was found for the “public transport story” (*M* = 3.52, *SD* = 0.92 vs. *M* = 2.33, *SD* = 1.01), *t*_(595.309)_ = 15.017, *p* < 0.001, *d* = 1.231, and for the “compulsory vaccination story” (*M* = 3.11, *SD* = 0.97 vs. *M* = 2.21, *SD* = 0.88), *t*_(597)_ = 11.829 *p* < 0.001, *d* = 0.967.

The perceived likelihood of a good ending of the news story was also higher when the participants read the text version that was intended to elicit the impression of a likely good (vs. bad) outcome. This effect was significant for the “public transport story” (*M* = 2.39, *SD* = 0.99 vs. *M* = 2.05, *SD* = 0.88), *t*_(587.250)_ = 4.482, *p* < 0.001, *d* = 0.367, and for the “compulsory vaccination story” (*M* = 2.79, *SD* = 0.97 vs. *M* = 2.25, *SD* = 0.87), *t*_(588.956)_ = 7.180 *p* < 0.001, *d* = 0.587.

The text version that was intended to increase participants' impression that the news story is of high personal relevance (home country) led to higher relevance ratings than the text version characterized by a low personal relevance (foreign county). This effect was found for the “public transport story” (*M* = 3.70, *SD* = 1.20 vs. *M* = 2.36, *SD* = 1.28), *t*_(595.339)_ = 13.142, *p* < 0.001, *d* = 1.074, and for the “compulsory vaccination story” (*M* = 3.20, *SD* = 1.13 vs. *M* = 2.10, *SD* = 1.12), *t*_(597)_ = 11.883 *p* < 0.001, *d* = 0.971.

As in Study 2, a higher personal relevance of a news story was not accompanied by a higher perceived utility of information. The manipulated relevance had only a marginally significant effect on perceived utility in the “public transport story” and no significant effect in the “compulsory vaccination story,” both *t*s ≤ 1.657, *p*s ≥ 0.098, *d*s ≤ 0.135. Importantly, on a descriptive level, the less personally relevant text version was perceived as more informative in both cases.

To conclude, the manipulations were successful without exception.

#### Do a high likeability of the protagonist (H1a) and a likely bad outcome (H1b) increase perceived suspense?

We initially calculated a 2 (manipulated likeability of the protagonist) × 2 (manipulated likelihood of a good ending) × 2 (manipulated relevance of news story) × 2 (news story) mixed-measures ANOVA with suspense as dependent variable.

The data did not support H1a and H1b, as shown in Figure 1G. We only found a significant main effect of the news story, *F*_(1, 591)_ = 40.361, *p* < 0.001, $\eta_{p}^{2}$ = 0.064. The “public transport story” (*M* = 2.74, *SD* = 0.89) elicited more suspense than the “compulsory vaccination story” (*M* = 2.51, *SD* = 0.84). No further effects reached statistical significance, all *F*s ≤ 2.418, *p*s ≥ 0.121, $\eta_{p}^{2}$ ≤ 0.004.

#### Does reading appreciation positively correlate with experienced suspense (H1c) and does the personal relevance of a news story moderate this correlation (RQ1)?

We computed a moderation analysis by using Model 1 of the PROCESS macro (Hayes, 2016). Reading appreciation served as dependent variable, suspense served as independent variable, and the manipulated relevance of the news story served as a moderator (dummy-coded: 0 = low relevance, 1 = high relevance). We used centered variables to avoid problems of multicollinearity. For the “public transport story,” the analysis revealed an *R*^2^ of 0.208, *F*_(3, 595)_ = 51.953, *p* < 0.001, whereby the experienced suspense showed a positive effect on reading appreciation, *B* = 0.513, *t* = 12.21, *p* < 0.001. The manipulated relevance of the news story showed a marginally significant effect, *B* = −0.128, *t* = −1.714, *p* = 0.087. Importantly, the interaction term was not significant, *B* = 0.055, *t* = 0.653, *p* = 0.514. The results were similar for the “compulsory vaccination story,” as the analysis revealed an *R*^2^ of 0.130, *F*_(3, 595)_ = 29.603, *p* < 0.001. Again, the experienced suspense showed a positive effect on reading appreciation, *B* = 0.424, *t* = 9.335, *p* < 0.001. The manipulated relevance of the news story, *B* = −0.050, *t* = −0.648, *p* = 0.518, and the interaction term showed no effect, *B* = −0.056, *t* = −0.620, *p* = 0.535. Hence, experienced suspense positively correlated with reading appreciation, supporting H1c once more. This relationship was not moderated by the manipulated relevance of the news story, contradicting RQ1.

#### Does lingering interest positively correlate with experienced suspense (H1d) and does the personal relevance of a news story moderate this correlation?

We again ran the moderator analysis, whereby the lingering interest in the news story served as dependent variable. For the “public transport story,” we found an *R*^2^ of 0.242, *F*_(3, 595)_ = 63.351, *p* < 0.001, whereby the experienced suspense showed a positive effect, *B* = 0.670, *t* = 13.727, *p* < 0.001. We found no effect of the manipulated relevance of the news story, *B* = 0.032, *t* = 0.369, *p* = 0.712, and no effect of the interaction term, *B* = −0.094, *t* = −0.961, *p* = 0.337. For the “compulsory vaccination story,” we found an *R*^2^ of 0.183, *F*_(3, 595)_ = 44.376, *p* < 0.001, whereby the experienced suspense, *B* = 0.559, *t* = 11.285, *p* < 0.001, and the manipulated relevance of the news story, *B* = 0.231, *t* = 2.775, *p* = 0.006, showed a positive effect. Again, the interaction term did not reach statistical significance, *B* = −0.043, *t* = −0.435, *p* = 0.664. Consequently, these data are another support for H1d.

#### Is the expected correlation between participants' liking of the protagonist and reading appreciation (H2a)/lingering interest (H2b) mediated by the intensity of experienced suspense?

**Figure 2:**
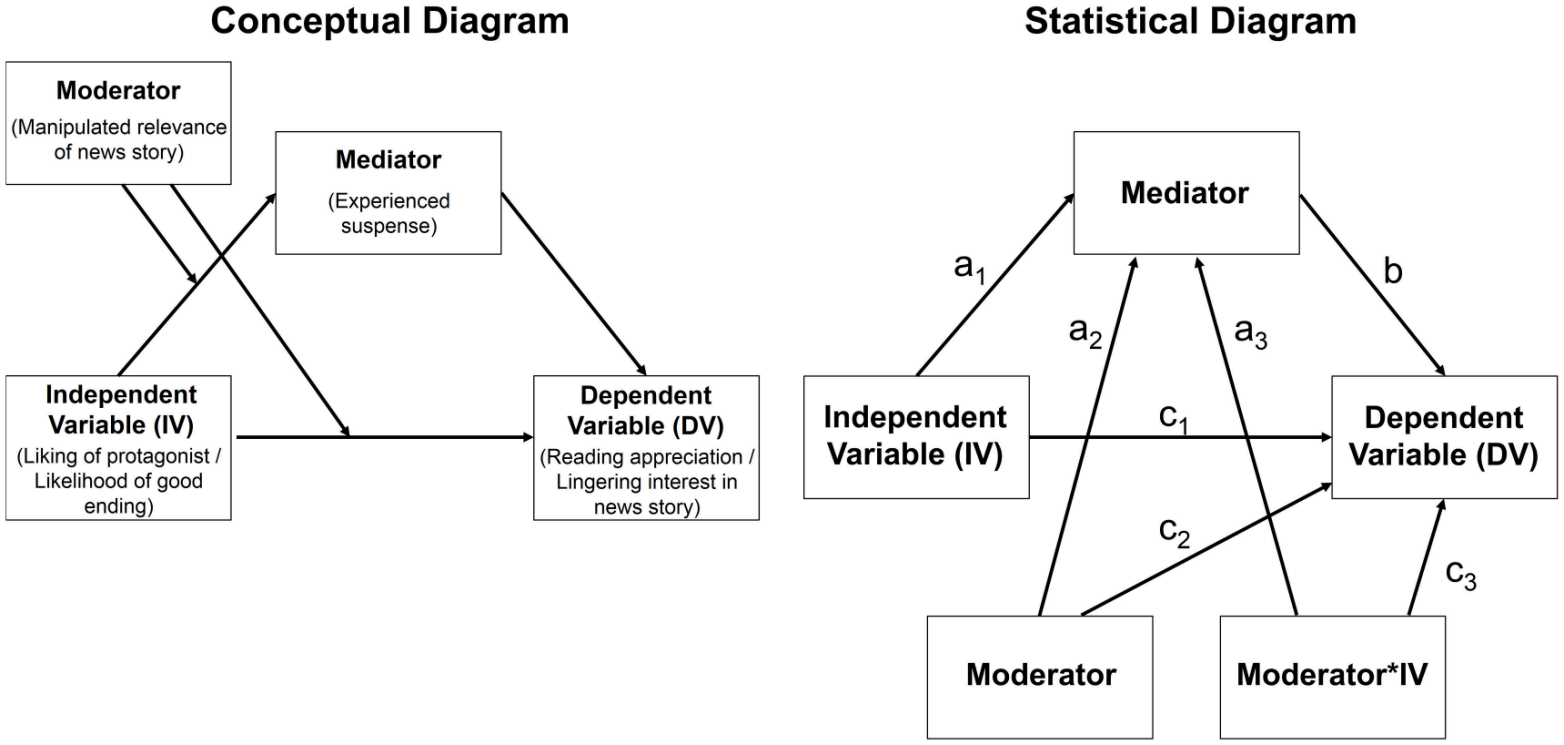
**The conceptual and statistical diagrams for the (moderated) mediation model tested in Study 3 (H2a–H2d)**.

We tested the mediation hypotheses by using Model 8 of the PROCESS macro (Hayes, 2016). The mediation model examined in Studies 1 and 2 was extended by the manipulated personal relevance of the news story (dummy-coded: 0 = low relevance, 1 = high relevance) which served as an additional moderator variable (for the conceptual and statistical diagram of the moderated mediation model, see Figure 2).

As shown in Table 5, we found positive correlations between the reported liking of the protagonist and suspense, between suspense and reading appreciation/lingering interest, and between the reported liking of the protagonist and reading appreciation/lingering interest. Importantly, the latter correlation was (partially) mediated by suspense as indicated by tests of indirect effects. This mediation process was independent of the manipulated personal relevance of the news story, indicating no moderated mediation. The result pattern was found for both news stories. Additionally, with respect to the “compulsory vaccination story,” the manipulated relevance showed an effect on lingering interest as more personally relevant news led to higher lingering interest.

**Table 5:** **Analyses of moderated mediation models of Study 3 (H2a and H2b)**.

	**Public transport story**				**Compulsory vaccination story**			
**Pathway**	***Coeff***.	***SE***	***t***	***p***	***Coeff***.	***SE***	***t***	***p***
**H2a**								
Model summary (criterion = mediator)	*R^2^* = 0.051, *F*_(3, 595)_ = 10.566, *p* < 0.001^***^				*R^2^* = 0.030, *F*_(3, 595)_ = 6.171, *p* < 0.001^***^			
Effect of IV on mediator (*a_1_*)	0.164	0.031	5.215	<0.001^***^	0.140	0.033	4.184	<0.001^***^
Effect of moderator on mediator (*a_2_*)	−0.106	0.071	−1.492	0.136	−0.051	0.068	−0.749	0.454
Effect of IV^*^moderator on mediator (*a_3_*)	−0.083	0.063	−1.315	0.189	0.037	0.067	0.549	0.583
Model summary (criterion = DV)	*R^2^* = 0.392, *F*_(4, 594)_ = 95.756, *p* <0.001^***^				*R^2^* = 0.322, *F*_(4, 594)_ = 70.576, *p* <0.001^***^			
Effect of mediator on DV (*b*)	0.409	0.038	10.837	<0.001^***^	0.337	0.041	7.270	<0.001^***^
Effect of IV on DV ($c_{1}^{\prime }$)	0.396	0.030	13.393	<0.001^***^	0.430	0.034	12.775	<0.001^***^
Effect of moderator on DV ($c_{2}^{\prime }$)	−0.106	0.066	−1.617	0.106	−0.002	0.068	−0.034	0.973
Effect of IV^*^moderator on DV ($c_{3}^{\prime }$)	0.039	0.058	0.667	0.505	−0.046	0.067	−0.685	0.493
Conditional indirect effect of IV on DV at moderator value = low relevance	effect = 0.084 (*SE_*boot*_* = 0.019), 95%*CI*: 0.048–0.124^*^				effect = 0.041 (*SE_*boot*_* = 0.017), 99%*CI*: 0.0001–0.088^**^			
Conditional indirect effect of IV on DV at moderator value = high relevance	effect = 0.050 (*SE_*boot*_* = 0.021), 95%*CI*: 0.011–0.092^*^				effect = 0.053 (*SE_*boot*_* = 0.018), 99%*CI*: 0.011–0.106^**^			
Index of moderated mediation	index = −0.034 (*SE_*boot*_* = 0.027), 90%*CI*: −0.080–0.010				index = 0.012 (*SE_*boot*_* = 0.023), 90%*CI*: −0.025–0.052			
**H2b**								
Model summary (criterion = DV)	*R^2^* = 0.308, *F*_(4, 594)_ = 66.129, *p* < 0.001^***^				*R^2^* = 0.193, *F*_(4, 594)_ = 35.529, *p* < 0.001^***^			
Effect of mediator on DV (*b*)	0.601	0.048	12.574	<0.001^***^	0.536	0.050	10.724	<0.001^***^
Effect of IV on DV ($c_{1}^{\prime }$)	0.277	0.037	7.395	<0.001^***^	0.115	0.041	2.774	0.006^**^
Effect of moderator on DV ($c_{2}^{\prime }$)	0.048	0.083	0.579	0.563	0.244	0.083	2.938	0.003^**^
Effect of IV^*^moderator on DV ($c_{3}^{\prime }$)	0.117	0.073	1.598	0.111	0.042	0.082	0.519	0.604
Conditional indirect effect of IV on DV at moderator value = low relevance	effect = 0.123 (*SE_*boot*_* = 0.027), 95%*CI*: 0.071–0.178^*^				effect = 0.065 (*SE_*boot*_* = 0.026), 95%*CI*: 0.017–0.118^*^			
Conditional indirect effect of IV on DV at moderator value = high relevance	effect = 0.074 (*SE_*boot*_* = 0.030), 95%*CI*: 0.016–0.135^*^				effect = 0.085 (*SE_*boot*_* = 0.027), 95%*CI*: 0.033–0.141^*^			
Index of moderated mediation	index = −0.050 (*SE_*boot*_* = 0.040), 90%*CI*: −0.115–0.015				index = 0.020 (*SE_*boot*_* = 0.037), 90%*CI*: −0.041–0.081			

*H2a: IV = liking of protagonist, Mediator = experienced suspense, DV = reading appreciation, Moderator = manipulated relevance of news story*.

*H2b: IV = liking of protagonist, Mediator = experienced suspense, DV = lingering interest, Moderator = manipulated relevance of news story*.

* *p < 0.05*,

** *p < 0.01*,

*** *p < 0.001*.

*The table depicts the model summaries, unstandardized coefficients, and the point estimate for the (moderated) indirect effects*.

#### Is the expected correlation between the perceived likelihood of a good ending of a news story and reading appreciation (H2c)/lingering interest (H2d) mediated by the intensity of experienced suspense?

With respect to H2c and H2d, the results were completely identical with those reported above, as shown in Table 6. Overall, both the reported liking of the protagonist (H2a and H2b) and the perceived likelihood of a positive ending (H2c and H2d) showed a direct and an indirect effect on reading appreciation and lingering interest. Thus, experienced suspense was a significant mediator.

**Table 6:** **Analyses of moderated mediation models of Study 3 (H2c and H2d)**.

**Pathway**	**Public transport story**				**Compulsory vaccination story**			
	***Coeff***.	***SE***	***t***	***p***	***Coeff***.	***SE***	***t***	***p***
**H2c**								
Model summary (criterion = mediator)	*R^2^* = 0.031, *F*_(3, 595)_ = 6.355, *p* < 0.001^***^				*R^2^* = 0.030, *F*_(3, 595)_ = 6.192, *p* < 0.001^***^			
Effect of IV on mediator (*a_1_*)	0.158	0.039	4.028	<0.001^***^	0.148	0.036	4.147	<0.001^***^
Effect of moderator on mediator (*a_2_*)	−0.046	0.072	−0.623	0.534	−0.035	0.068	−0.517	0.605
Effect of IV^*^moderator on mediator (*a_3_*)	−0.002	0.078	−0.019	0.985	0.038	0.072	0.531	0.595
Model summary (criterion = DV)	*R^2^* = 0.255, *F*_(4, 594)_ = 50.850, *p* < 0.001^***^				*R^2^* = 0.175, *F*_(4, 594)_ = 31.401, *p* < 0.001^***^			
Effect of mediator on DV (*b*)	0.471	0.041	11.399	<0.001^***^	0.382	0.045	8.510	<0.001^***^
Effect of IV on DV ($c_{1}^{\prime }$)	0.247	0.040	6.180	<0.001^***^	0.226	0.040	5.684	<0.001^***^
Effect of moderator on DV ($c_{2}^{\prime }$)	−0.017	0.075	−0.224	0.823	−0.002	0.075	−0.030	0.976
Effect of IV^*^moderator on DV ($c_{3}^{\prime }$)	0.007	0.079	0.091	0.928	−0.046	0.079	−0.584	0.560
Conditional indirect effect of IV on DV at moderator value = low relevance	effect = 0.075 (*SE_*boot*_* = 0.030), 95%*CI*: 0.019–0.135^*^				effect = 0.050 (*SE_*boot*_* = 0.021), 95%*CI*: 0.010–0.094*			
Conditional indirect effect of IV on DV at moderator value = high relevance	effect = 0.074 (*SE_*boot*_* = 0.026), 95%*CI*: 0.026–0.126^*^				effect = 0.064 (*SE_*boot*_* = 0.021), 95%*CI*: 0.027–0.108*			
Index of moderated mediation	index = −0.001 (*SE_*boot*_* = 0.038), 90%*CI*: −0.063–0.064				index = 0.015 (*SE_*boot*_* = 0.029), 90%*CI*: −0.032–0.063			
**H2d**								
Model summary (criterion = DV)	*R^2^* = 0.259, *F*_(4, 594)_ = 51.896, *p* < 0.001^***^				*R^2^* = 0.202, *F*_(4, 594)_ = 37.482, *p* < 0.001^***^			
Effect of mediator on DV (*b*)	0.641	0.049	13.104	<0.001^***^	0.528	0.050	10.624	<0.001^***^
Effect of IV on DV ($c_{1}^{\prime }$)	0.170	0.047	3.598	<0.001^***^	0.158	0.044	3.579	<0.001^**^
Effect of moderator on DV ($c_{2}^{\prime }$)	0.109	0.089	1.234	0.218	0.262	0.083	3.184	0.002^**^
Effect of IV^*^moderator on DV ($c_{3}^{\prime }$)	0.100	0.093	1.066	0.287	0.094	0.087	1.085	0.278
Conditional indirect effect of IV on DV at moderator value = low relevance	effect = 0.102 (*SE_*boot*_* = 0.040), 95%*CI*: 0.024–0.181^*^				effect = 0.068 (*SE_*boot*_* = 0.030), 95%*CI*: 0.012–0.129^*^			
Conditional indirect effect of IV on DV at moderator value = high relevance	effect = 0.101 (*SE_*boot*_* = 0.034), 95%*CI*: 0.037–0.171^*^				effect = 0.089 (*SE_*boot*_* = 0.028), 95%*CI*: 0.035–0.145^*^			
Index of moderated mediation	index = −0.001 (*SE_*boot*_* = 0.052), 90%*CI*: −0.086–0.084				index = 0.020 (*SE_*boot*_* = 0.039), 90%*CI*: −0.044–0.086			

*H2c: IV = perceived likelihood of a good ending, Mediator = experienced suspense, DV = reading appreciation, Moderator = manipulated relevance of news story*.

*H2d: IV = perceived likelihood of a good ending, Mediator = experienced suspense, DV = lingering interest, Moderator = manipulated relevance of news story*.

* *p < 0.05*,

** *p < 0.01*,

*** *p < 0.001*.

*The table depicts the model summaries, unstandardized coefficients, and the point estimate for the (moderated) indirect effects*.

#### Does a high (vs. low) manipulated likelihood of a good ending increase participants' reading appreciation only in the case of more (vs. less) personally relevant news stories (H3)?

The 2 × 2 × 2 × 2 ANOVA with reading appreciation as dependent variable only showed main effects for the news story (public transport story: *M* = 3.36, *SD* = 1.02; compulsory vaccination story: *M* = 2.83, *SD* = 1.00), the manipulated likeability of the protagonist (low: *M* = 2.92, *SD* = 0.72; high: *M* = 3.28, *SD* = 0.76, see Figure 1H), and the manipulated relevance of the news story (low: *M* = 3.16 *SD* = 0.75; high: *M* = 3.03, *SD* = 0.76), all *F*s ≥ 5.062, *p*s ≤ 0.025, $\eta_{p}^{2}$ ≥ 0.008. All other effects did not reach statistical significance, all *F*s ≤ 2.310, *p*s ≥ 0.129, $\eta_{p}^{2}$ ≤ 0.004. Hence, we did not find evidence for the interaction effect outlined in H3. Interestingly, reading appreciation was higher when the news story was of less personal relevance.

#### Effects of manipulated news characteristics on lingering interest

Finally, for the sake of completeness and in favor of a comparison with the results of the previous studies, we computed the 2 × 2 × 2 × 2 ANOVA with lingering interest as dependent variable. We found a main effect of the news story, *F*_(1, 591)_ = 100.320, *p* < 0.001, $\eta_{p}^{2}$ = 0.145, that was additionally qualified by its manipulated relevance, *F*_(1, 591)_ = 5.051, *p* = 0.025, $\eta_{p}^{2}$ = 0.008. The effect of the news story was larger when the manipulated relevance was low (public transport story: *M* = 3.17, *SD* = 1.20; compulsory vaccination story: *M* = 2.52, *SD* = 1.11, *t* = 8.848, *p* < 0.001) compared to when it was high (public transport story: *M* = 3.12, *SD* = 1.24; compulsory vaccination story: *M* = 2.71, *SD* = 1.13, *t* = 5.426, *p* < 0.001); but the effect of the news story was significant, independent of the manipulated relevance. Also, the manipulated likelihood of a good ending showed an effect, *F*_(1, 591)_ = 6.567, *p* = 0.011, $\eta_{p}^{2}$ = 0.011. As shown in Figure 1I, the prospect of a happy ending (*M* = 2.98, *SD* = 0.98) vs. bad ending (*M* = 2.78, *SD* = 0.96) increased the lingering interest, replicating the result of Study 2. No further significant effects occurred, all *F*s ≤ 2.395, *p*s ≥ 0.122, $\eta_{p}^{2}$ ≤ 0.004, except one marginally significant three-way interaction between the three between-subject factors (*p* = 0.090) that is not examined in detail here.

### Summary

The manipulations of the protagonist's likeability, the likelihood of a good ending, and the personal relevance of the news story were successful in both news stories. In contrast to Study 1, the protagonist's manipulated likeability and the likelihood of a good ending did not affect suspense, contradicting H1a and H1b. Supporting H1c and H1d, reading appreciation and lingering interest positively correlated with experienced suspense, while this relationship was not moderated by the manipulated relevance of the news story, contradicting our expectations formulated in RQ1. As in Studies 1 and 2, the type of news story did not influence all of these results. Hence, this result pattern found in Study 3 was more congruent with those found in Study 2 than those found in Study 1. Apparently, a perfect replication of KWK's original hypotheses was only achieved when the news stories had an inherently thrilling and drama-like potential (Study 1), questioning the general validity of the preconditions for suspense experience formulated by Zillmann (1996).

However, we cannot conclude that the news stories of Studies 2 and 3 produced more similar results overall. Rather, Study 3 revealed very mixed results regarding reading appreciation and lingering interest: in contrast to Study 2, but in accordance with Study 1, we did not find an effect of the manipulated likelihood of a good ending on reading appreciation, but a high vs. low (manipulated) likeability of the protagonist increased reading appreciation. In contrast to Study 1, but in accordance with Study 2, participants' lingering interest in the news story was increased in the case of a high vs. low (manipulated) likelihood of a good ending, whereas the protagonist's manipulated likeability did not affect lingering interest. Thus, the effect of manipulated news characteristics appear to be rather idiosyncratic and tied to specific news topics.

Moreover, Study 3 was intended to answer whether the manipulated likelihood of a good ending of a news story and its manipulated relevance show an interaction effect on reading appreciation (H3). The data did not support this interaction hypothesis. We only found a main effect of the news story's personal relevance, whereby reading appreciation was higher when the news story was of less personal relevance. This result is surprising but might be explained by the fact that the personally more relevant version of the story implied consequences for the recipients being not fully desirable, such as an additional tax obligation (public transport story) or a visit to the doctor (compulsory vaccination story).

Furthermore, we again observed a considerable discrepancy between effects related to manipulated vs. actually perceived news characteristics: while effects of the manipulated likeability of the protagonist and the likelihood of a good ending on suspense, reading appreciation, and lingering interest were sparse or even completely absent (see above), participants' actual liking of the protagonist and the perceived likelihood of a good ending showed positive correlations with all these measurements. This result once more proposes to conceptually differentiate between manipulated and perceived news characteristics. Also, the observed correlations again contradict the assumption that likely negative outcomes are beneficial for suspense experiences and the enjoyment of news stories, as postulated by the affective-disposition theory in the context of fictional dramas (cf. Zillmann, 1996).

Finally, both the reported liking of the protagonist and the perceived likelihood of a good ending showed not only a direct but also an indirect effect on reading appreciation as well as lingering interest, indicating that suspense is a significant mediator and supporting H2. Hence, the mediation effects found in Study 2 and (partially) found in Study 1 were replicated. We therefore conclude that on the level of perceived news characteristics, suspense reliably mediates the enjoyment of news stories.

## Conclusion

The present work investigated the role of experienced suspense in the context of news stories. It has been claimed that suspense may not only be a significant determinant of the enjoyment of a narrative in the context of entertainment media, but that it may also be a core variable when consuming news reports (Knobloch-Westerwick and Keplinger, 2007). According to Zillmann's affective-disposition theory (Zillmann, 1996), there are two critical characteristics of fictional dramas determining suspense: the likeability of a protagonist and the likelihood of a bad ending. If both variables are high, one may experience a high level of suspense. We transferred this model to news stories by manipulating their content accordingly. Moreover, it has been claimed that suspense positively influences the enjoyment of a news story indicated by one's reading appreciation and lingering interest in the news story (Knobloch-Westerwick and Keplinger, 2007). Hence, suspense was expected to be a significant mediator. However, we also hypothesized that suspense may act differently in the context of low vs. high personally relevant news stories. Across three studies, we examined these assumptions and found several important results:

First of all, we were able to reliably manipulate the likeability of the protagonist and the likelihood of a good ending (Studies 1, 2, and 3) as well as the personal relevance of a news story (Study 3). Interestingly, we observed a large absence of interaction effects between these news characteristics across all three studies. Also, the type of news story did not substantially interact with the manipulated news characteristics, but it scaled the absolute values of some dependent variables. Given the multifactorial designs, this is a bit surprising. In more positive terms, we may conclude that the different characteristics of a news story can be independently varied to a large degree. However, one should note that the present news stories were completely invented, whereas reports about real-world situations may (and should) limit the degrees of freedom for editorial manipulations.

In accordance with a previous study (Knobloch-Westerwick and Keplinger, 2007), Study 1 showed that the manipulated likeability of the protagonist and the manipulated likelihood of a good ending affected suspense. In contrast, Studies 2 and 3 did not support a significant effect of the manipulated news characteristics on suspense. Hence, it appears that the validity of Zillmann's model, which was originally developed to explain suspense in the context of fictional dramas, is limited in the context of news stories. Note that we used news stories characterized by an inherently thrilling and drama-like potential in Study 1, whereas we used news about political issues and debates in Studies 2 and 3. Furthermore, the effects of the manipulated news characteristics on reading appreciation and lingering interest in the news story were inconsistent across all three studies. Therefore, we may conclude that the systematic variation of central news characteristics does not produce reliable effects on recipients' emotional responses and news perception.

However, across all three studies, we found positive correlations between experienced suspense and reading appreciation as well as lingering interest. This result supports the claim that suspense does not only positively influence the enjoyment of fictional dramas (Zillmann, 1996) but also the enjoyment of news stories (Knobloch-Westerwick and Keplinger, 2007). So, one may conclude that media producers are confronted with a dilemma: on the one hand, suspense reliably promotes the enjoyment of news stories, but, on the other hand, the manipulation of critical news characteristics does not necessarily boost suspense.

However, things were very different when it came to the perceived news characteristics. Indeed, the conceptual and methodological distinction between manipulated news characteristics and participants' actual impressions of the news was important. While effects of the manipulated likeability of the protagonist and the likelihood of a good ending on suspense, reading appreciation, and lingering interest were inconsistent and sometimes absent across the three studies, participants' actual liking of the protagonist and the actually perceived likelihood of a good ending showed relatively stable positive correlations with all these measurements. Moreover, the correlation between the actually perceived news characteristics and reading appreciation/lingering interest was consistently mediated by suspense, while this mediation was sometimes partial and sometimes complete. Importantly, the personal relevance of the news stories, which was systematically manipulated in Study 3, neither moderated this mediation process nor the correlation between suspense and reading appreciation/lingering interest. Overall, the personal relevance did not show substantial effects.

In a nutshell, we may conclude that suspense significantly mediates the correlation between perceived news characteristics and the enjoyment of news stories, whereas theory-driven manipulations of news characteristics do not necessarily influence the enjoyment of narratives as desired. We may speculate that the latter variability reflect different types of emotion targeted by the stories (e.g., fear or compassion in Study 1 vs. anger in Study 2) as well as emotional intensity, modulating the emotional relevance of the news characteristics for suspense and the enjoyment of the stories. In this sense, suspense and, more generally, emotional responses to news stories appear to be a critical factor in news perception and should hence be part of a broader research strategy in the field of news research. Therefore, we finally want to point out some avenues for future research:

First, we used single items as dependent variables, except for the suspense scale. In general, it might be desirable to develop multi-item measurements for perceived news characteristics, emotional responses, and news perception. Research, however, is almost a blank page in this regard. Therefore, and for the sake of replication, we used the one-item scales created by KWK. Nonetheless, it is conceivable that some of the constructs have several facets, making multi-item scales necessary. But one could also argue that recipients make a final judgment about their affective disposition toward the protagonist or about the liking of a news story, justifying one global item. Also, a multi-item scale may be heavily tied to specific features of specific news, threatening its general validity. Thus, the conceptual differentiation remains an open question for future research.

Second, the present studies may be limited regarding the generalizability of the findings to other cultures because we only investigated the effects in samples of German participants being comparable in terms of age, gender, education, and current job position. As KWK also focused on German students, an attempt to replicate these effects in other cultures would be desirable. At least some evidence exists that proposes cross-cultural differences in media use (e.g., Kononova and Chiang, 2015), in the interpretation of identical information (e.g., Bente et al., 2014), in post-receptive effects of news consumption (e.g., Facorro and DeFleur, 1993), and in news coverage by media (e.g., Iyengar et al., 2009). Also, it has to be noted that the present samples of participants were characterized by a strong gender bias, including about 75–80% females. The random assignment of participants to experimental conditions prevented confounds in the results, but it is conceivable that the observed values were affected in absolute terms as gender differences has been reported regarding media use (Ohannessian, 2009) and emotional responses to media content (Peck, 2000).

Third, it appears noteworthy that the actually perceived likelihood of a good vs. bad ending was found to increase suspense, reading appreciation, and lingering interest in Studies 2 and 3. This result indicates a preference for happy endings (Ross and Simonson, 1991) and it contradicts the notion that likely negative outcomes are beneficial for suspense and the enjoyment of news stories, as postulated by the affective-disposition theory in the context of fictional dramas (cf. Zillmann, 1996). Future studies might scrutinize whether participants current mood state moderates this effect. For example, a positive mood sometimes increases one's preference for negative news content (Kaspar et al., 2015b).

Finally, future research should expand our focus to more long-term effects of suspense on reading appreciation and lingering interest. It is conceivable that one's lingering interest in a news story fluctuates over time and that the liking of news reports that only add incremental insights to the plot may show a gradual decrease until the final “showdown.” This implies repeated-measures designs over a substantial period of time and shifts the attention away from the status-quo in this research field.

## Author contributions

KK conceived and designed the experiments. KK and DZ contributed materials and performed the experiments. KK performed the data analysis. KK and AW interpreted the data and wrote the manuscript. DZ approved the final version of the manuscript for submission.

### Conflict of interest statement

The authors declare that the research was conducted in the absence of any commercial or financial relationships that could be construed as a potential conflict of interest.
