# Supplementary material for: Thrilling News Revisited: The Role of Suspense for the Enjoyment of News Stories
Source: Front Psychol. 2016 Dec 15;7:1913. doi: 10.3389/fpsyg.2016.01913 (PMC5156838; doi:10.3389/fpsyg.2016.01913)
Supplement: Supplementary file 1 [file Presentation1.PDF]

Thrilling news revisited:
The role of suspense for the enjoyment of news stories

Kai Kaspar, Daniel Zimmermann, & Anne-Kathrin Wilbers

Supporting Information File S1

This supplementary file presents the fictitious news stories used in Studies 1, 2, and 3.
Words depicted in *italics* have been manipulated according to the experimental condition.
Blue color indicates the words manipulated in favor of a positive/negative affective disposition toward the protagonist.
Red color indicates the words manipulated in favor of a high/low likelihood of a good ending.
Green color indicates the manipulated personal relevance of the news story.

Study 1 – News Story “Accident”

News scenario with a positive affective disposition toward the protagonist and a high likelihood of a good ending:

Original German version used in the study

"Nach einen Autounfall auf einer Landstraße nahe Ismaning bei München wurde Maria Nuckertid (34) ins Krankenhaus eingeliefert. *Sie wurde durch ihre Spendenorganisation „Alle Kinder Sind Gleich“, welche Familien von behinderten und pflegebedürftigen Kindern finanziell unterstützt, medial bekannt.* Laut Augenzeugen kam ihr Auto aufgrund von Blitzeis von der Straße ab und prallte dabei frontal auf einen Baum. Ein Zeuge kontaktierte daraufhin umgehend den Notdienst, zog die Bewusstlose aus dem Auto und leistete erste Hilfe, bis der Krankenwagen eintraf. Maria Nuckertid wurde noch am Unfallort von den Notärzten behandelt und später im Krankenhaus operiert. Laut des behandelnden Arztes der unfallchirurgischen Station habe Maria Nuckertid ein Schädel-Hirn-Trauma erlitten und sei derzeit noch bewusstlos. *Mehrere Untersuchungen haben dabei ergeben, dass Ihr Zustand aktuell stabil und nicht mehr lebensbedrohlich sei. Laut des Arztes werde die Patientin mit großer Wahrscheinlichkeit sehr bald aus dem Koma erwachen.* Im Moment befindet sich Maria Nuckertid auf der Intensivstation und wird von ihrem Lebensgefährten umsorgt. Weitere Untersuchungen und Aktualisierungen zu ihrem Zustand werden in den kommenden Tagen folgen, versicherte der Sprecher des Krankenhauses."

English translation

"After a car accident on a rural road close to Ismaning, Munich, Maria Nuckertid (34) was brought to hospital. *She gained fame for her charity organization “All children are equal” which gives financial support to families with disabled children*. Eye witnesses reported that her car went off the street due to flash freeze and then frontally bumped into a tree. One witness directly called the ambulance, pulled the unconscious person out of the car, and gave first aid until the ambulance arrived. Maria Nuckertid was treated by the emergency doctor at the accident location and later on operated in hospital. The attending doctor of the trauma surgery ward declared that she suffered from a craniocerebral injury and is still unconscious. *Several examinations revealed that her condition is stable and not life-threatening anymore. As per her doctor, there is a high probability that she will awake from coma very soon.* At the moment, Maria Nuckertid is in intensive care being shepherded by her partner. Further examinations and updates will follow in the next days, the speaker of the hospital assured."

In the news scenario with a negative affective disposition toward the protagonist it says:

Sie wurde durch die Verwahrlosung und Misshandlung ihrer drei Kinder und dem folgenden Streit mit dem Jugendamt medial bekannt. / She gained fame for a mistreatment and squalidness of her three children and a resulting fight with the youth welfare service.

In the news scenario with a low likelihood of a good ending it says:

Mehrere Untersuchungen haben dabei ergeben, dass Ihr Zustand aktuell instabil und immer noch lebensbedrohlich sei. Laut des Arztes werde die Patientin mit großer Wahrscheinlichkeit nicht mehr aus dem Koma erwachen. / Several examinations revealed that her condition is still unstable and life-threatening. As per her doctor, there is a high probability that she will not awake from coma.

Study 1 – News Story “Kidnapping”

News scenario with a positive affective disposition toward the protagonist and high likelihood of a good ending:

Original German version used in the study

"Nach der Freilassung von zwei französischen Journalisten in Niger wurde ein weiterer französischer Staatsbürger in Bolivien entführt. Bei dem Mann soll es sich laut französischen Medien um Mathéo Linagesev (39) handeln, *einem Mitglied der Hilfsorganisation "Ärzte ohne Grenzen", der ehrenamtlich in einem Krankenhaus im Norden Boliviens arbeitet und während eines Außeneinsatzes* von vier bewaffneten Männern an Bord eines Geländewagens gezogen und verschleppt worden sei. Eine dort bekannte politisch-motivierte Guerilla-Gruppe hat sich bereits zu dem Angriff bekannt und fordert im Austausch mit dem Gefangenen die Freilassung von zwei inhaftierten Mitgliedern ihrer Gruppe. In der Vergangenheit machte sich diese Gruppe schon häufiger durch Entführungen von Journalisten und Mitgliedern von Hilfsorganisationen auf sich aufmerksam, forderte bisher jedoch lediglich die Zahlung von Lösegeld. Dabei kam noch niemand ums Leben, alle Gefangenen wurden bisher wieder freigelassen. Französische Geheimdienste haben sich bereits eingeschaltet, um mit der bolivianischen Regierung das Vorgehen zur Befreiung Mathéo Linagesevs zu besprechen. *Diese zeigt sich bisher bereit, mit den Guerilla zu verhandeln und auf die Forderungen einzugehen. Daher halten französische Medien eine baldige Rettung des 39-Jährigen für wahrscheinlich.*"

English translation

"After the release of two French journalists in Niger, another French statesman was kidnapped in Bolivia. French media supposes that the kidnapped man is Mathéo Linagesev (39), *a member of the charity organization “doctors without borders”, who voluntarily works in a hospital in the north of Bolivia. During an outdoor mission*, he was pulled into an off-road vehicle by four armed men and displaced. A locally famous politically motivated guerilla group already admitted the attack and demands the exchange of two arrested members of their group to free the captive. In the past, this group already called attention to itself by hijacking of journalists or members of charity organizations but up to now, they only demanded ransom. Until now, no one has lost their lives and all captives were released. French secret services already stepped in to discuss with the Bolivian government the course of action to free Mathéo Linagesev. *The government already showed willingness to negotiate with the guerrilla and to agree to their demands. French media reckon that a quick rescue of the 39-year old is likely.*"

In the news scenario with a negative affective disposition toward the protagonist it says:

...einem Journalisten, der in Frankreich aufgrund eines von ihm betriebenen rechtsextremen Internetblogs stark in mediale Kritik geriet und während einer Reportagetour... / ...a journalist who was massively criticized in media for his right-wing internet blog. During a working trip...

In the news scenario with a low likelihood of a good ending it says:

Diese zeigt sich bisher jedoch nicht bereit, mit der Guerilla zu verhandeln oder auf ihre Forderungen einzugehen. Daher halten französische Medien eine baldige Rettung des 39-Jährigen für unwahrscheinlich. / The government is not willing to negotiate with the guerrilla or to agree to their demands. French media reckon that a quick rescue of the 39-year old is unlikely.

Study 2 – News Story "Fair working conditions"

News scenario with a positive affective disposition toward the protagonist and a high likelihood of a good ending:

Original German version used in the study

"Die Arbeitsbedingungen für junge Menschen verschlechtern sich zunehmend. Ein Beispiel ist Torsten Freiholdt (28), dessen Fall kommende Woche vor dem Arbeitsgericht verhandelt wird. Nach zweijähriger Vollzeitbeschäftigung in einem großen Unternehmen hoffte er, eine unbefristete Festanstellung zu erhalten. Das Unternehmen möchte ihn hingegen nicht weiter beschäftigen aufgrund „grundlegender Veränderungen der Personalstruktur". Zukünftig sollen alle unter 30-jährigen Arbeitnehmer des Unternehmens nur noch auf maximal zwei Jahre befristet angestellt werden. Dagegen hat Torsten Freiholdt geklagt, denn für ihn ist der Ausgang von existentieller Bedeutung. *Vor seiner Zeit beim jetzigen Arbeitgeber war er drei Jahre als Entwicklungshelfer in Afrika tätig* und konnte in dieser Zeit keinerlei finanzielle Rücklagen ansparen. Der Ausgang des Verfahrens ist aber nicht nur für Torsten Freiholdt relevant. Vielmehr sehen Personalexperten in dem Verfahren eine Signalwirkung. Wenn das Unternehmen für seine Personalpolitik durch das Arbeitsgericht Recht zugesprochen bekäme, könnten auch andere Unternehmen ähnlich verfahren, um Personalkosten generell reduzieren oder flexibel einsparen zu wollen. Es steht also nicht nur für Torsten Freiholdt viel auf dem Spiel. *Insgesamt, so Personalexperten, seien die Chancen, dass Torsten Freiholdt das Verfahren gegen das Unternehmen gewinnen wird, äußerst groß.*"

English translation

"The working conditions for young people are decreasing tremendously. An example for this is Torsten Freiholdt (28), whose case will be heard at labor court next week. After a two-year full-time job at a large company, he hoped to get a permanent contract. But the company is not willing to pursue his engagement due to "basic structural changes in their human resources". In future, all employees under 30 should receive a fixed-term work contract for two years only. Torsten Freiholdt brought this to trial, because the result is of existential importance for him. *Before he started working at his present employer, he was a development aid worker in Africa* and wasn´t able to save money for a financial reserve. The result of this trial is not only meaningful to Torsten Freiholdt. Human resources experts expect a signaling from this trial. If the company wins this trial with its human resources policy, other companies might act in the same manner to reduce personnel costs flexibly or to save them in general. Thus, the stakes are high, not only for Torsten Freiholdt. *All in all, human resources experts see high chances for him to win the trial.*"

In the news scenario with a negative affective disposition toward the protagonist it says:

Vor seiner Zeit beim jetzigen Arbeitgeber war er drei Jahre aufgrund einer Vorbestrafung wegen Diebstahls arbeitssuchend… / Before he started working at his present employer, he was seeking work for three years due to his criminal record as a thief…

In the news scenario with a low likelihood of a good ending it says:

Insgesamt, so Personalexperten, seien die Chancen, dass Torsten Freiholdt das Verfahren gegen das Unternehmen gewinnen wird, äußerst gering. / All in all, human resources experts see only minimal chances for him to win the trial.

Study 2 – News Story "Affordable housing"

News scenario with a positive affective disposition toward the protagonist and a high likelihood of a good ending:

Original German version used in the study

"Es gibt in den Großstädten Deutschlands immer weniger Möglichkeiten für Geringverdiener, eine bezahlbare Wohnung zu finden. So lässt sich auch für Matthias Hörschler (24) aus Bremen weder ein WG-Zimmer noch eine für ihn finanzierbare Wohnung finden. *Er möchte nach dreijähriger freiwilliger sozialer Arbeit in einem Altersheim ein Architektur-Studium in Köln beginnen.* Da Hörschler nur in Köln einen Studienplatz bekam, wird er nun gerichtlich vorgehen und reicht Klage beim Land NRW ein, um zu erwirken, dass Städte und Kommunen einen bestimmten Prozentsatz an Wohnungen zur Verfügung stellen müssen, die für Geringverdiener bezahlbar sind. Der Ausgang des Verfahrens werde laut Miet- und Wohnungsexperten nicht nur für NRW richtungsweisend, vielmehr werde dies auch bundesweite Auswirkungen haben. So gebe es in vielen Metropolenregionen und Universitätsstädten einen Mangel an entsprechender Wohnfläche. Sollte das Verfahren zugunsten des Studenten ausgehen, könne man sich darauf einstellen, dass es in naher Zukunft in ganz Deutschland zwangsweise mehr günstigere Wohnungen für Studenten, Auszubildenden und anderen Geringverdienern geben müsse. Sollte die Klage jedoch scheitern, werde dem weiteren Anstieg der Mietpreise durch die Preisspirale Tür und Tor geöffnet, warnen Verbraucherschützer. *Insgesamt sehen juristische Experten die Chance, dass Matthias Hörschler gewinnen und es zu einer Bereitstellungspflicht von günstigem Mietraum kommen wird, als sehr groß an.*"

English translation

"In large German cities are less and less possibilities for low-income earners to find an affordable flat. Also for Matthias Hörschler (24) it is impossible to find an affordable flat or room in a shared-flat. *After three years of social work in a home for elderly people, he wants to study architecture in Cologne.* Because he only received a university place in Cologne, he wants to institute proceedings against the land of North Rhine-Westphalia to obtain that cities have to provide a certain percentage of houses for low-income earners. Housing- and rental experts assume that the result of this trial will not only have effects for North Rhine-Westphalia but also German wide. In most large cities and university cities is a lack of affordable living space. If the trial results in favor of the student, it might be possible that all over Germany very soon affordable flats for students, trainees and low income earners become a must. But if the complaint fails, an increase of rental prices will be given, consumer protectors warn. *All in all, law experts see high chances for Matthias Hörschler to win the trial and to introduce an appropriation obligation for cheap rented premises.*"

In the news scenario with a negative affective disposition toward the protagonist it says:

Er möchte nach dreijährigem Aufenthalt in einer Jugendvollzugsanstalt ein Architektur-Studium in Köln beginnen. / After three years of juvenile arrest, he wants to study architecture in Cologne.

In the news scenario with a low likelihood of a good ending it says:

Insgesamt sehen juristische Experten die Chance, dass Matthias Hörschler gewinnen und es zu einer Bereitstellungspflicht von günstigem Mietraum kommen wird, als sehr klein an. /
All in all, law experts see only minimal chances for Matthias Hörschler to win the trial and to introduce an appropriation obligation for cheap rented premises.

Study 3 – News Story "Public transport"

News scenario with a positive affective disposition toward the protagonist, a high likelihood of a good ending, and high personal relevance:

Original German version used in the study

„Die Benutzung öffentlicher Verkehrsmittel wie Bus und Bahn ist ein politisches Dauerthema in *Deutschland*. Nun scheint es einen neuen Versuch zu geben, das Bus- und Bahnfahren für alle bezahlbar zu machen. Vielmehr noch: Es steht die Idee im Raum, auf den Verkauf von Fahrscheinen komplett zu verzichten. Finanziert werden soll der öffentliche Verkehr *in Deutschland* stattdessen über eine ans individuelle Einkommen angepasste Pflichtsteuer, die zukünftig jeder steuerzahlende Bürger des Landes abgeben soll. Einfach in Bus und Bahn einsteigen und mitfahren – ohne Fahrticket und völlig legal. So lautet die Grundidee der Initiative von Eduard Moellen (55), der einen entsprechenden Antrag mit Unterstützung mehrerer Politiker und großer medialen Aufmerksamkeit nun ins *deutsche* Parlament geben will. Eduard Moellen ist schon zuvor medial in Erscheinung getreten als ehemaliger Vorstand *einer Hilfsorganisation für Menschen in Not und mehrmals ausgezeichneter Politiker*. Nun also versucht er, das öffentliche Verkehrswesen nachhaltig zu verändern. Experten aus Politik und Wirtschaft schätzen dabei die Wahrscheinlichkeit, dass Eduard Moellen mit seiner neuen Initiative erfolgreich sein wird, als *sehr hoch* ein."

English translation

"The use of public transport such as bus and train is a permanent political topic in *Germany*. Now there seems to be a new attempt to make the bus and train travel affordable for everyone. Rather than that, the idea of a complete abdication of the sale of tickets is introduced. Instead, public transport *in Germany* will be funded by a tax obligation adapted to the individual income which will be paid by each tax-paying citizen of the country in the future. Getting on buses and trains and just ride – legally without having a ticket. This is the main idea of the initiative of Eduard Möllen (55) who wants to give an application to the *German* Parliament with the support of multiple politicians and under great media attention. Eduard Möllen has previously made his medial appearance as a former director of *a charity organization for people in need as well as a politician who has been awarded several times*. So now he's trying to change the public transport system sustainably. Experts from politics and industry evaluate the likelihood that Eduard Möllen will be successful with his new initiative as *very high*."

In the news scenario with a negative affective disposition toward the protagonist it says:
einer radikalen und mittlerweile verbotenen Partei und mehrfach angeklagter Politiker / a radical and now prohibited party as well as a repeatedly accused politician

In the news scenario with a low likelihood of a good ending it says:
sehr gering / very low

In the news scenario with low personal relevance we substituted *Germany/German* by *Norway/Norwegian* (or adjective)

Study 3 – News Story "Compulsory vaccination"

News scenario with a positive affective disposition toward the protagonist, a high likelihood of a good ending, and high personal relevance:

Original German version used in the study

„Die Debatte um eine Impfpflicht in *Deutschland* ist wieder entfacht. Immer wieder treten Krankheiten in der Bevölkerung auf, die durch eine rechtzeitige und einfache Impfung leicht verhindert werden könnten. Offenbar machen existierende Impfempfehlungen die Notwendigkeit bestimmter Impfungen nicht ausreichend deutlich. Insbesondere *deutsche* Erwachsene scheinen des Öfteren notwendige Impfungen zu übersehen oder einfach zu ignorieren. Der Arzt Manuel Köpbergen (48) hat nun einen Verband gegründet, dessen Ziel die Einführung einer umfassenden Impfpflicht für Kinder und Erwachsene in *Deutschland* ist. Manuel Köpbergen war selbst zuvor jahrelang Hersteller von Medikamenten. Er kam *dabei positiv in die Schlagzeilen aufgrund seiner wohltätigen Arbeit in Afrika und der Unterstützung von vielen hilfsbedürftigen Patienten durch kostenlose Medikamente*. Die von ihm nun gestartete Initiative zur Schaffung einer Impfpflicht in *Deutschland* kommt nun langsam ins Rollen und würde die Nachimpfung fast aller Erwachsenen bedeuten, da auch neuartige Impfungen vorgenommen werden sollen. Gesundheitsexperten und Politiker sind sich ziemlich sicher, dass diese Initiative *mit hoher* Wahrscheinlichkeit eine Impfflicht tatsächlich herbeiführen kann."

English translation

"In *Germany*, the debate about compulsory vaccination has aroused again. Over and over again, diseases occur in the population that could be easily prevented by a timely and simple vaccination. Apparently, existing vaccination recommendations do not sufficiently underline the need for certain vaccinations. Especially *German* adults seem to overlook necessary vaccinations or simply ignore them frequently. The physician Manuel Köpbergen (48) has now established an association that aims for introducing a compulsory vaccination for children and adults in *Germany*. Manuel Köpbergen himself had previously been a producer of drugs. Thereby, he made *positive headlines due to his charity work in Africa and the support of many needy patients by free medical products*. His initiative aims for the establishment of a compulsory vaccination in *Germany* and it is gaining momentum now. This initiative would lead to a revaccination of almost all adults, because novel vaccines should also be applied. Health experts and politicians are quite sure that this initiative may actually induce a compulsory vaccination *with high* probability."

In the news scenario with a negative affective disposition toward the protagonist it says:
allerdings negativ in die Schlagzeilen aufgrund unseriöser Wirksamkeitsversprechen und der finanziellen Ausnutzung von hilfsbedürftigen Patienten / negative headlines due to his dubious efficacy promises and the financial utilisation of needy patients

In the news scenario with a low likelihood of a good ending it says:
nur mit geringer / with only low

In the news scenario with low personal relevance we substituted *Deutschland/Germany* by *Dänemark/Denmark* (or adjective)
